# Biomechanics of the Peacock’s Display: How Feather Structure and Resonance Influence Multimodal Signaling

**DOI:** 10.1371/journal.pone.0152759

**Published:** 2016-04-27

**Authors:** Roslyn Dakin, Owen McCrossan, James F. Hare, Robert Montgomerie, Suzanne Amador Kane

**Affiliations:** 1 Department of Zoology, University of British Columbia, Vancouver, Canada; 2 Drexel University, Philadelphia, PA, United States of America; 3 Department of Biological Sciences, University of Manitoba, Winnipeg, Canada; 4 Department of Biology, Queen's University, Kingston, Canada; 5 Physics Department, Haverford College, Haverford, PA, United States of America; University of Sussex, UNITED KINGDOM

## Abstract

Courtship displays may serve as signals of the quality of motor performance, but little is known about the underlying biomechanics that determines both their signal content and costs. Peacocks (*Pavo cristatus*) perform a complex, multimodal “train-rattling” display in which they court females by vibrating the iridescent feathers in their elaborate train ornament. Here we study how feather biomechanics influences the performance of this display using a combination of field recordings and laboratory experiments. Using high-speed video, we find that train-rattling peacocks stridulate their tail feathers against the train at 25.6 Hz, on average, generating a broadband, pulsating mechanical sound at that frequency. Laboratory measurements demonstrate that arrays of peacock tail and train feathers have a broad resonant peak in their vibrational spectra at the range of frequencies used for train-rattling during the display, and the motion of feathers is just as expected for feathers shaking near resonance. This indicates that peacocks are able to drive feather vibrations energetically efficiently over a relatively broad range of frequencies, enabling them to modulate the feather vibration frequency of their displays. Using our field data, we show that peacocks with longer trains use slightly higher vibration frequencies on average, even though longer train feathers are heavier and have lower resonant frequencies. Based on these results, we propose hypotheses for future studies of the function and energetics of this display that ask why its dynamic elements might attract and maintain female attention. Finally, we demonstrate how the mechanical structure of the train feathers affects the peacock’s visual display by allowing the colorful iridescent eyespots–which strongly influence female mate choice–to remain nearly stationary against a dynamic iridescent background.

## 1. Introduction

Courtship displays often involve movements that stimulate multiple senses [1–5]. Research on such multimodal courtship displays has focused primarily on what they can tell us about patterns in the evolution and diversification of sexually selected traits, e.g., [1, 5, 6]. Much less is known about the biomechanics of display performance [7], even though it has been proposed that dynamic displays may serve as signals of the quality of motor performance [8, 9]. An understanding of the energetic costs, muscle power limits, and biomechanical constraints underlying display performance is necessary to evaluate what these displays might be signaling (Clark 2012) and thus to understand their function [9].

**Fig 1:**
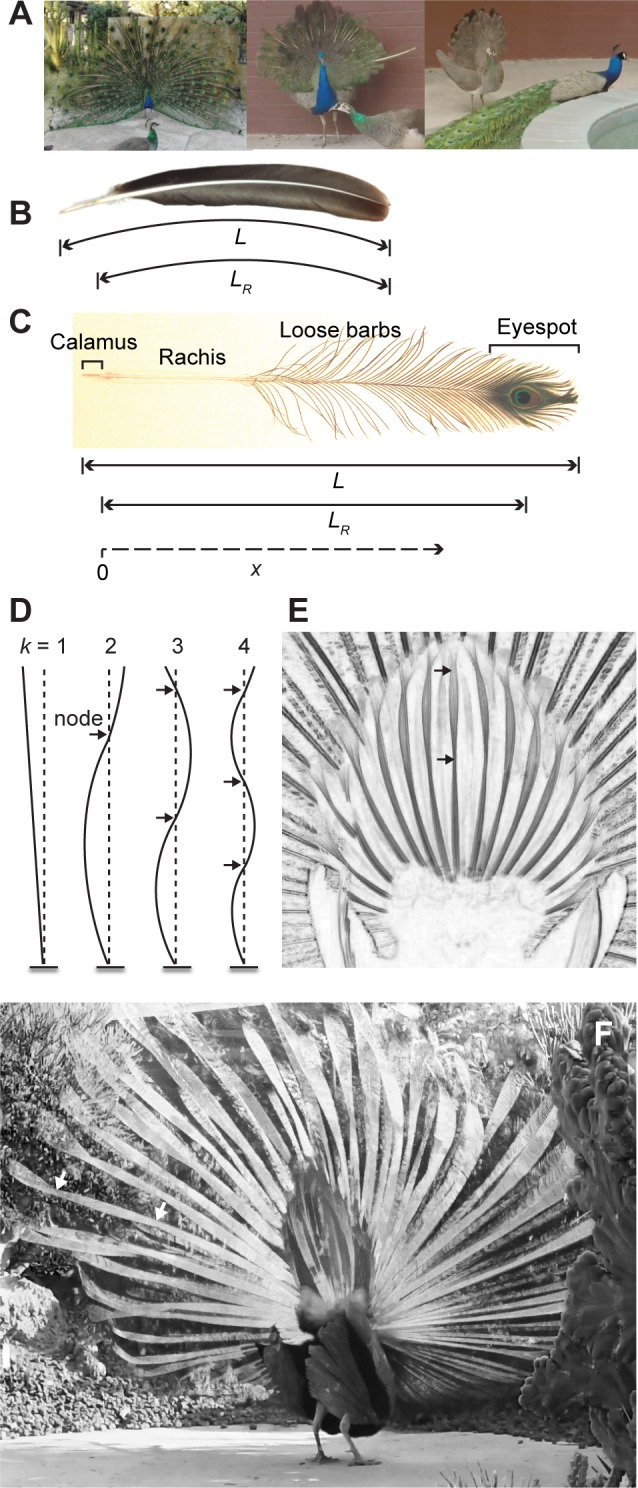
Peafowl displays and feather anatomy. (A) Photos (left to right) show an adult male, a subadult male, and a female performing train-rattling (males) or covert-rattling (female) displays. Feather anatomy and measurements taken from (B) a typical adult peacock rectrix (tail feather) and (C) an eyespot train feather. The length of the rachis outside the body (i.e., excluding the calamus), *L*_*R*_, is used to characterize the mechanics of vibrations. The position along the rachis, *x*, is measured from the insertion point into the skin (top of the calamus) as shown. (D) Examples of normal modes of oscillation for a cantilever with a fixed tip, indexed by the integer *k*, where *k* = 1 corresponds to the lowest resonant frequency. For a given pattern of vibration corresponding to a normal mode, the value of *k* can be determined by counting the number of nodes (i.e., points of zero motion, indicated with arrows). (E) Motion of tail feathers viewed from behind a peacock during the train-rattling display. A video showing 10 cycles of oscillation during train-rattling was converted to high-contrast grayscale and then the individual frames were superimposed to show the entire range of motion of the rachises during vibrations. The resulting image illustrates the similarity between the cantilever normal modes shown in D and the patterns of feather rachis vibrations. For example, the centermost tail feather (rectrix) has two nodes (arrows) and is thus oscillating in the *k* = 3 normal mode. (F) Similar image to E illustrating the motion of train feather rachises viewed from the rear during train-shivering. Arrows indicate that the locations of two nodes in one of the "fishtail" train feathers.

Indian peafowl (*Pavo cristatus*) have a complex courtship display wherein males (peacocks) engage females (peahens) with elongated train (upper tail covert) feathers that display iridescent colors [10–12] and make mechanical sounds (S1 Movie) [13]. A courting peacock will raise his tail and train feathers (Fig 1A), and often vibrates those feathers once the peahen is in front of him [14]. This “train-rattling” display attracts the peahen’s visual attention [15] and always precedes copulation [14]. Most of the train feathers have a single eyespot at the distal end, and peacocks that display eyespots with greater iridescence obtain more matings [11, 12], whereas natural variation among peacocks in train length and the number of displayed eyespots is not associated with mating success [10].

In this study, we used a combination of field studies and *in vitro* experiments to characterize the biomechanics of the peacock’s train-rattling courtship displays. Our aim was to describe natural variation in this display and to evaluate how performance is influenced by the morphology and mechanical properties of the feathers. We used high-speed video analysis to determine the pattern of movement of vibrating feathers, focusing on how the eyespots can appear to hover motionless against a background of oscillating iridescent feather parts, a feature that has been suggested to enhance the peacock’s visual display [16]. We also analyzed audio recordings of the mechanical sounds produced to investigate how bioacoustic properties of the sounds relate to the frequencies of feather vibrations.

We then compared peacock train-rattling displays to the shaking behaviors of other animals. The vibration frequency of other behaviors, such as shivering and shaking dry, scales negatively with body mass across species [17–20]. One possible explanation for this negative scaling is that, all else being equal, the power required to drive vibrations (drive power) increases with both the vibration frequency and the mass shaken [17, 21]. If peacock displays are determined by similar mechanical and physiological constraints, we expect the average feather vibration frequency during the peacock’s train-rattling display to agree with the values predicted for these other behaviors.

Another factor that may influence peacock shaking behavior is the resonant properties of peacock feathers. Like any elastic mechanical system, the peacock’s train should have its greatest vibrational amplitude when driven near a resonant frequency of oscillation [22]. As a complex system with many degrees of freedom, the train should have multiple resonant frequencies at which mechanical power is most efficiently transferred from the drive source [21]. This means that for a given shaking amplitude, the peacock should be able to use the least muscular power if it shakes the train at a resonant frequency. However, not all animal vibrations are performed at resonance (e.g., insect wing flapping [23]). We therefore investigated how peacock display vibration frequencies compare with peacock feather resonant frequencies.

At resonance, a mechanical structure also vibrates in a characteristic spatial pattern called a normal mode in which the nodes and antinodes (points of zero and maximum motion, respectively) are stationary. Because feathers are a type of cantilever beam (i.e., a bar anchored at one end), their vibrational motions at resonance should resemble the normal modes of a cantilever. We studied whether feather motion during displays was consistent with cantilever normal modes.

Variation among peacocks with respect to their morphological traits (body size, feather length, etc.) may also influence the feather vibrational frequency used for train-rattling. For example, train length varies considerably among peacocks–and this trait is highly correlated with train mass [24]–so train length should determine vibrational frequency for two reasons. First, peacocks with heavier trains should require more power to vibrate their feathers at a given frequency and amplitude. Thus, as one would expect based on other animal shaking behaviors, individuals with longer and heavier trains should use lower vibration frequencies to minimize costs. Second, we expected that train length would influence the resonant properties of the train. Cantilever beam theory predicts that each normal mode’s resonant frequency should decrease rapidly with increasing feather length, *L* [21]. For both of these reasons, longer-trained peacocks could potentially reduce energy costs by vibrating their trains at lower frequencies.

Building on our field studies, we performed *in vitro* measurements to characterize the resonant properties of peacock feathers. With lengths up to 1.5 m, each train feather weighs less than 2 g, yet stands erect with minimal support and endures hundreds of hours of display every breeding season (spanning 2–3 months). The eyespot feathers in the train also vary in length by an order of magnitude, so cantilever beam theory predicts that different feathers within the train should have resonant frequencies that vary by more than an order of magnitude [21, 25]. This raises the question of how peacocks can efficiently drive their trains at a single vibrational frequency. To determine how resonant frequencies of peacock train and tail feathers depend on their length, we performed shaking experiments on individual feathers and feather arrays similar to those used to study resonance in the feathers of other species [26, 27].

Next we determined the range of vibration frequencies that can be used to efficiently drive feathers near resonance; this also characterizes their damping (energy dissipation). Systems with low damping are in resonance only over a narrow range of frequencies. In contrast, systems with high damping have the ability to vibrate efficiently over a wider range of frequencies. Therefore, low damping results in more efficient coupling of muscle power to train motion, but only at specific frequencies, whereas high damping has the advantage of allowing the displaying male to vibrate his train and tail feathers efficiently at a wide range of frequencies [21].

Finally, we combined our laboratory and field data to evaluate whether the vibration frequency of train-rattling agrees with the resonant frequencies found for train and tail feathers. While this study focuses on the proximate biomechanical mechanisms of peacock displays, we discuss how our results may guide future studies of display function and evolution. We also discuss our findings in the context of other types of feathers and elongated biological structures, such as whiskers, where function depends upon resonant properties.

## 2. Methods

### 2.1 Field Methods

We studied feral peafowl in Arcadia, California, USA (34.1416° N, 118.0538° W) from 10–24 March 2015. Courtship activity in this study population peaks from early March to early April [12]. We obtained hourly air temperature from the KCQT, CA weather station (34.0235°N, 118.2912°W) (data obtained online at https://www.wunderground.com/history/airport/KEMT/2016/03/30/DailyHistory.html?req_city=Arcadia&req_state=CA&reqdb.zip=91006&reqdb.magic=1&reqdb.wmo=99999 and accessed 8 November 2015) to include ambient air temperature in some analyses.

Courtship displays were observed from 07:30–11:00 and 15:00–18:30 PDT. Individual adult males (defined as males displaying a full train > 1 m long) were identified by their fidelity to a display court, whereas subadult males (displaying shorter, incomplete trains) and females were individually recognizable by their unique morphological features (i.e., tail, face, and crest plumage).

Video recordings were made with a Hero4 Black camera (240 frames s^-1^ non-interlaced; 720 × 1280 pixels; GoPro, San Mateo, CA, USA) using a 2X VLC252B telephoto lens (Bower Lenses, Long Island City, NY, USA). We video-recorded the train-rattling displays performed by 14 adult peacocks, 14 subadult peacocks, and five peahens that performed a similar behavior that we call “covert-rattling”, in which the peahens vibrated their tails and upper tail coverts (Fig 1A; S2 Movie).

We also studied two other shaking behaviors performed by adult peacocks whether or not females were present: “wing-shaking” (S1 Movie), in which males shake their wings behind the erect train [14], and “train-shivering” (S3 Movie), in which peacocks spread or reposition their train feathers into a fan [13]. Sample sizes for all behaviors are provided in Table 1. Train-rattling displays were recorded from each peacock at different times and on different days. Although we recorded every female covert-rattling display that we observed, the sample size is small because this behavior is rare.

**Table 1:** Feather vibration frequencies (Hz) during peafowl shaking behaviors.

behavior	no. of bouts (no. of individuals)	grand mean (Hz)* [95% confidence interval]	range of individual means (Hz)
*train- or covert-rattling*			
adult male	40 (14)	25.6 [25.0, 26.1]	23.9–27.1
subadult male	32 (14)	26.5 [25.7, 27.3]	24.9–29.2
female	8 (5)	26.1 [25.3, 27.0]	25.2–27.1
*train-shivering*			
adult male	18 (7)	10.4 [10.1, 10.6]	9.8–10.7
*wing-shaking*			
adult male	24 (11)	5.4 [4.9, 5.9]	4.5–6.8

*Grand means were calculated as the mean of individual (bird) means at peak display amplitude.

We made audio recordings of the train-rattling displays of 10 adult peacocks using a PMD661 recorder (±1 dB: 20 Hz to 24 kHz; Marantz, New York, NY, USA) and a ME-62 microphone (±2.5 dB: 20 Hz to 20 kHz; Sennheiser, Wedemark, Germany) with a K-6 powering module at 44.1 kHz and 24-bit depth with no filtering.

To investigate associations between peacock morphology and vibration frequency, we measured morphological traits for 12 adult males (two video-recorded males were not measured because they displayed on rooftops). Tail length, defined as the contour length of the longest rectrix (tail feather), was measured by taking a video of a ruler held touching the erect tail of each displaying male. Train length was measured from video images as the contour length of the longest train feathers in the middle of the train relative to tail length. For analysis, train length was corrected for an estimated growth rate of 0.41 cm day^-1^ during the period of our study (S1 Text). The number of eyespots displayed in the train was counted on frontal images of the raised train.

All methods and sampling procedures were reviewed and approved by the University of British Columbia Animal Care Committee (Animal Utilization Protocol A15-0037) and the Los Angeles County Arboretum and Botanical Garden. This study was conducted with permission of the Los Angeles Country Arboretum and Botanical Garden.

### 2.2 Comparison to Other Animal Shaking Behaviors

Previous research indicates that the frequencies animals use for some types of shaking behaviors are constrained, such that they scale (as a power law) with variation in body mass across species [17, 18]. To evaluate whether the vibrational frequencies in the peacock’s courtship display fit the prediction for one of these scaling laws, we compared them to the data and scaling relationships for animals both shivering [18] and shaking dry [17, 19, 20]. We also compared the vibration frequencies of peafowl displays with those of other avian courtship displays (S2 Text).

### 2.3 Audio Analyses

To analyze the mechanical sounds generated by feather motions during train-rattling, we used Raven Pro 1.4 [28] to generate audio spectrograms (Hann filter; 0.01 s time window for short time Fourier transform; 86.1 Hz frequency resolution; 95% time-window overlap). To determine the frequencies of maximum power emission during train-rattling, waveforms from three intense bouts of train-rattling by each of 10 adult males were Fourier analyzed to produce power spectra (Hann filter; 1.00 s time window; 1 Hz frequency resolution). We used these data to identify *n* = 116 peak values to determine the rate at which the “rattle” sounds were produced during train-rattling (S3 Text).

### 2.4 Feather Morphometrics and Vibrational Resonance

We studied the properties of molted adult male eyespot feathers and rectrices, and female tail covert feathers obtained from commercial sources and zoos. See Fig 1B and 1C for feather anatomy. The part of the feather’s central shaft or rachis that is inserted into the body is called the calamus. Rectrices have uniform, tightly meshed barbs along their length, whereas eyespot feathers have loose iridescent green barbs symmetrically distributed along the length of the feather, with close-packed barbs near the distal end that form the iridescent eyespot. We used a flexible ruler to measure both contour length of the entire feather, *L*, and its rachis length outside the body, *L*_*R*_ (Fig 1B and 1C), Rachis length *L*_*R*_ is the effective cantilever length and is proportional to feather length *L*.

We also measured the morphological properties of eyespot feathers that influence their mechanical integrity and resonant properties. To function properly during displays, the eyespot feathers must stand erect. Consequently, we computed the critical stresses at which these feathers would undergo Euler buckling (elastic bending through a large angle) and local buckling (the formation of irreversible kinks in the tube wall), using measurements of the elastic modulus, the dimensions, and the cross-sectional second moment of area, *I*, of the rachis.

The resonant frequencies, *f*_*k*_, of a cantilever with rachis length *L*_*R*_ and linear density of the feather (mass per unit length) *μ*_*F*_ are given by:

$$f_{k}=K_{k}^{2}\frac{1}{2\pi L_{R}^{2}}\sqrt{\frac{G'I}{\mu_{F}}}$$ (1)

The bending stiffness of the feather is the product of *I* (the rachis second moment of area) and *G*´ (the dynamic storage modulus) [29]. We indexed the normal modes of vibration using *k* = 1, 2, 3, etc. The shapes of the normal modes (Fig 1D) and their frequencies are determined by mechanical parameters of the feather–elasticity, inertia, geometry, and size–as well as any constraints on its motion. The unitless coefficient *K*_*k*_^*2*^ for each mode is determined by the boundary constraints on the tip (i.e., whether the tip is free to move, clamped or hinged), and any variation of *I*, *G’*, and *μ*_*F*_ with position along the length of the feather [29]. For the simplest case of a vibrating string, the normal modes are harmonic standing waves with resonant frequencies equal to integer multiples of the *k* = 1 (fundamental) frequency (i.e., the values of *K*_*k*_^*2*^ are integers). For a thick cantilever, *K*_*k*_^*2*^ takes on non-integer values such that the frequencies of the higher order normal modes are not integer multiples of the fundamental (*k* = 1) frequency.

We made measurements at various positions, *x*, along the rachis, where *x* is the distance from the base (insertion point) (Fig 1B and 1C). To measure the linear density of each feather, we cut it into segments of length *Δx* and measured the mass *ΔM* of each segment, with and without loose barbs, and then computed linear density for each segment as *μ*_*R*_ = *ΔM*_*R*_*/Δx* for the rachis and *μ*_*F*_ = *ΔM*_*F*_*/Δx* for the entire feather (i.e., the rachis and its barbs). To find *I(x)*, we also measured the rachis diameter *D* at each position *x* (± 0.03 mm) using digital calipers. To compute *I(x)* from *D(x)* and *μ*_*R*_*(x)*, each rachis segment was modeled as a rigid, cylindrical, pith-filled cortex shell [30] using known rachis densities and cross-sectional geometries (S4 Text). We measured the mass and length of the region encompassing the eyespot (including the adjacent close-packed barbs) to find the linear density of the eyespot for each feather. All measurements were performed on eyespot feathers of five different lengths *L*_*R*_ ≈ 14, 30, 66, 90, and 112 cm, and replicated for four feathers at each length.

To determine eyespot ultrastructure, eyespots were removed from one train feather from each of three peacocks and mounted on carbon conductive tape overlying a metal SEM stub. Different regions within each eyespot were imaged at magnifications from 40 to 300X using a TM-1000 Tabletop SEM (Hitachi Corporation, Tokyo, Japan).

Laboratory shaking experiments were performed on feathers to measure vibrational resonant responses (S4 Movie). In the field videos, train and tail feathers were observed to vibrate primarily in the lateral plane (parallel to the barbs), although displaying peacocks perform other slower, non-vibrational motions (i.e., tilting and turning the train; S1 Movie). Consequently, to study feather vibrations in the laboratory, vertically oriented feathers were subjected to low amplitude (< 2 mm) horizontal vibrations in the lateral plane using a mechanical shaker, and we confirmed that minimal out-of-plane motions occurred during these measurements. We used a model SF-9324 mechanical shaker (Pasco Scientific, Roseville, CA, USA) for all measurements except for those made on an array of train feathers shaken using the more powerful model K2007E01 shaker (Modal Shop, Sharonville, OH, USA). The shaking apparatus was rigidly mounted using optical posts on an optical honeycomb breadboard table (Thorlabs, Newton, NJ, USA) to eliminate extraneous vibrations in the frequency range of interest. We measured the vibrational response of the shaker head to be broadband, reproducible, and independent of feather length. All feather vibrational response spectra were normalized by the vibrational response of the shaker head (see 2.6 Data Analysis for further details).

A model 330120A function generator (Agilent Technologies, Wilmington, DE, USA) was used to drive the shaking apparatus. We used three different frequency sweep rates to allow sufficient time for equilibration of the shaking amplitude: 0.042 Hz s^-1^ over 0.5–3.0 Hz; 0.25 Hz s^-1^ over 0–15 Hz; and 1.8 Hz s^-1^ over 10–120 Hz. These frequency sweep parameters were chosen based on analysis of data from field videos indicating that at the beginning of the display the train-rattling frequency increases exponentially to a maximum (initial rate = 43 Hz s^-1^ ± 10 Hz s^-1^ measurement precision; time constant = 0.8 s [95% CI: 0.4, 1.2]). A constant FFT magnitude was reached after 0.27 s ± 0.04 s (measurement precision), shorter than the time taken for equilibration in the field videos. FFT magnitudes were not reproducible above ~40–60 Hz because at high frequencies the broadband noise due to discretization of the motion tracked on the video exceeded the amplitude of the shaker drive. We also obtained vibrational spectra for examples of each feather type while they were shaken at a constant frequency, and confirmed that the feathers only vibrated at the frequencies already in the range of frequencies produced by the shaker.

We tracked feather motion during vibrations using the same 240 frames s^-1^ video methods described in 2.1 above. Motion was tracked using either the eyespot or a white marker (< 6 mg) at the feather tip. While scanning laser vibrometry is appropriate for measuring out-of-barb-plane displacements, the train-rattling and train-shivering vibrations of interest occur in the plane of the eyespot and loose barbs. This geometry causes the outer loose barb motion to obscure the lateral motion of the rachis and eyespots, making it difficult to probe lateral vibrations of these features with this technology.

We used the shaking apparatus to characterize the resonant properties of single eyespot feathers (*L*_*R*_ ≈ 14, 30, 68, 90, 112 cm) and rectrices (*L*_*R*_ ≈ 27, 38, 45, 50 cm) with lengths similar to those measured on displaying peacocks in the field, as well as on single tail covert feathers from peahens (*L*_*R*_ ≈ 29 cm). We used 3 feathers of each of (i) the longest, shortest, and middle eyespot feather lengths, (ii) the longest rectrix length, and (iii) the peahen upper tail coverts (one feather was used for the other lengths). Each feather was mounted with its calamus embedded in a rigid plastic holder with hot glue; several feathers were also tested in a viscoelastic gel mount to mimic soft tissue. The gel mount had minimal effect on shaking spectra below 36–40 Hz (S5 Text). We also measured the vibrational response of arrays of each feather type to assess the interactions among feathers. We created three arrays: one array of 5 peacock rectrices, one array of 5 peahen upper tail coverts, and one array of 25 peacock train feathers (see S5 Text for details). Arrays were mounted in either a balsa wood block (peacock rectrix array, peahen tail covert array) or a viscoelastic gel (train array). For each array and for one of each feather length and type, the shaker was started from rest three times to confirm that values of *f*_*k*_, *Q*_*k*_, and transfer functions were reproducible below 40–60 Hz. To understand why the eyespots remain stationary during displays, we repeated these measurements on one eyespot feather (*L*_*R*_ = 111 cm) after adding a 68 mg mass to the eyespot using tape, and again after cutting off the eyespot (removing the eyespot and tape). Further details are provided in S5 Text.

### 2.5 Image Analysis

Video and still images were corrected for lens distortion using the Camera Calibration module in the Machine Vision toolbox of Matlab 2014a (MathWorks, Natick, MA, USA). We measured the vibration frequency in field videos by counting frames for 10 cycles of oscillation, after checking a few samples using Fourier analysis to establish that there were no higher harmonics. The start and end of a display bout were defined by the bird raising and lowering its train (male) or covert (female) feathers. Train- and covert-rattling displays were sampled at three non-overlapping intervals in each display bout: 10 cycles at peak amplitude, 10 cycles before the peak interval began, and 10 cycles after the peak interval ended (pre- and post-peak intervals could not be obtained from bouts shorter than 30 cycles of vibration). We also measured the change in feather vibration frequency at the onset of train-rattling for six adult males to check equilibration times and frequency sweep rates for the subsequent *in vitro* feather shaking experiments.

For three sequences of intense bouts of train-rattling for each of six adult males, we used the Fiji implementation of ImageJ [31] to track and measure the motion of eyespots and loose barbs that were vibrating at peak amplitude. Amplitudes were converted to mm using the average diameter of the bronze part of the eyespots (40.5 mm) for scale, to compare with peafowl visual acuity (S6 Text).

To determine the spatial pattern of feather vibrations during train-rattling and train-shivering, we used videos that showed a close-up, stable, rear view of the peacock’s tail and train. Images from each video were converted to high-contrast grayscale and superimposed for one or more cycles of oscillation. This resulted in an image that displayed the entire range of positions of the feather rachises during vibrations (e.g., Fig 1E). By examining these images, we were able to determine whether the rachises exhibited stationary nodes and antinodes consistent with the cantilever normal modes of vibration (e.g., Fig 1D and 1E). We then counted the number of stationary nodes along the rachis to determine the normal mode index *k* (because the number of nodes is equal to *k*– 1; Fig 1D). For cases where we could not form a stable set of superimposed images, we noted whether the nodes were located at fixed positions along the rachises. The same method was used on feathers shaken during our laboratory measurements to determine their spatial patterns of motion and normal mode indices.

To measure feather motion during *in vitro* feather-shaking experiments, we used ImageJ and Matlab to track the shaker head and the eyespot or feather tip. Autocontrast enhancement and thresholding were applied to extract features for automatic tracking. For a few eyespot feathers, we also tracked the motion of the rachis to check the frequency of low amplitude resonant peaks.

### 2.6 Data Analysis

To analyze vibration frequencies recorded in the field, we fit linear mixed-effects models (LMEM) with a Gaussian error distribution using R 3.2.1 [32]. We accounted for nonindependence of repeated samples from the same sampling units by entering display bout nested within individual identity as a random effect.

To evaluate the fit of alternative models to predict the vibration frequency of feathers during the train-rattling displays of adult peacocks, we ranked all models using the corrected Akaike’s Information Criterion (AICc), a measure of the relative goodness-of-fit. Models with ∆AICc ≤ 2 (compared to the best-fitting model) were considered equally likely given the data. As a measure of the variance explained by the fixed effect predictors, we estimated R^2^_GLMM(m)_ for models with ∆AICc ≤ 2 [33]. Details of all candidate models in this analysis are provided in S7 Text.

We also determined repeatability, or the proportion of variation attributable to differences among adult males, using the variance components from an intercept-only mixed-effects model of peak vibration frequencies during train-rattling (*n* = 40 samples from 14 adult males) following [34].

To analyze the motion of feathers during the shaking experiments, we computed spectrograms in Matlab, which accounted for the variation in frequency. The resulting magnitudes, *A*, of the fast Fourier transform (FFT) at each vibrational drive frequency, *f*_*d*_, were divided by the corresponding shaker drive magnitude, *A*_*d*_, at that frequency to give the drive transfer function (*A/A*_*d*_), and then smoothed over a 1.3 Hz window using a cubic Savitzky-Golay filter. Each *k*^th^ peak in the transfer function was fitted to a Lorentzian function to obtain the resonant frequency, *f*_*k*_, and quality factor, *Q*_*k*_ [21]. The quality factor *Q*_*k*_ characterizes damping in resonant systems, and is defined as

$$Q_{k}\equiv 2\pi \frac{\text{energy stored}/\text{cycle}}{\text{energy dissipated}/\text{cycle}}=\frac{f_{k}}{\Delta f_{k,3dB}}.$$ (2)

The value of *Δf*_*k*,*3dB*_ describes the effective width of a resonant peak because the corresponding drive power falls to half its peak value at a distance ± *Δf*_*k*,*3dB*_ from the resonant peak at *f*_*k*_ [21]. In general, good resonators with weak damping have *Q* > 2π and proportionately narrow peaks. Resonators with strong damping have ½ < *Q* < 2π and proportionately wider resonant peaks.

We measured spectral peak widths, *Δf*_*k*,*3dB*_, directly from the response spectra for data that were not well approximated by a Lorentzian function. Because feather length and the position at which motion was tracked varied among samples, we scaled the transfer function for each feather to have a maximum value of 1, except when we added mass to, and then removed the eyespot from, a single eyespot feather. In this latter case, we directly compared the magnitudes of transfer functions because motion was always tracked at either the eyespot or the tip of that feather after the eyespot was removed.

To model the resonant properties of the entire tail (rectrices only), we estimated the drive power required to shake an array of rectrices with lengths estimated from (i) our peacock morphology data, (ii) mean rectrix linear densities measured directly on single feathers, and (iii) resonant frequencies derived from our shaking experiments on single feathers. Further details are provided in S8 Text.

Other computations and nonlinear least-squares fits were performed using either Origin 8.0 (OriginLab) or Matlab. Results are reported as mean [95% confidence interval]. All data and statistical analyses are provided in S1 Datasets and S9 Text.

## 3. Results

### 3.1 Peafowl Shaking Behaviors

During train-rattling displays, peacocks courted peahens by shaking their rectrices laterally (i.e., side to side) against the erect train at a mean frequency of 25.6 Hz (*n* = 14 displays by 14 adult peacocks; Table 1). The motion starts with the tail and spreads to the train via frictional coupling (S1 Movie), with a constant frequency reached within 2.4 s [1.2, 3.6] of onset (*n* = 6 displays by 6 adult males).

### 3.2 Comparison to Other Animal Shaking Behaviors

**Fig 2:**
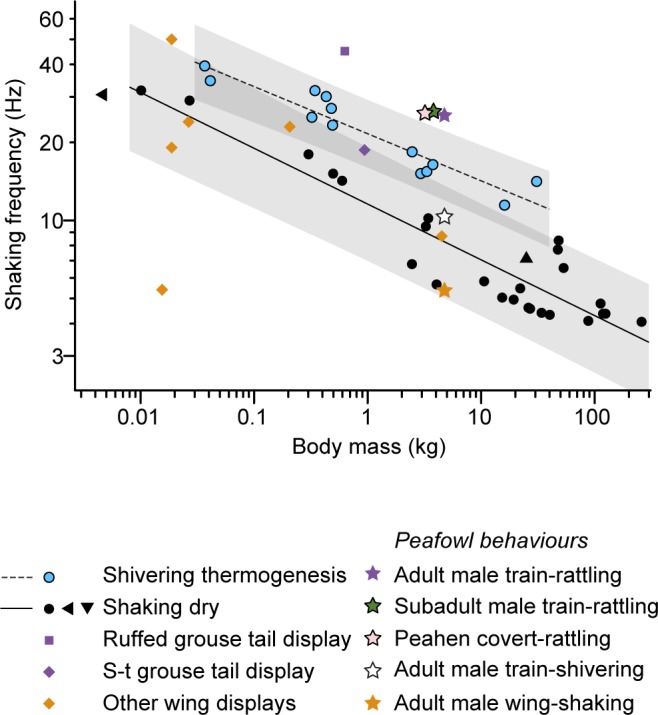
The average feather-shaking frequency of the peacock’s train-rattling display is above the predictions for an animal the mass of a peacock shivering and shaking dry, respecitvely. The mean values for peafowl behaviors are denoted with stars. Lines indicate fitted power law models derived in allometric studies of other shaking behaviors (Kleinebeckel and Klugmann 1990) (Dickerson, Mills and Hu 2012) with shaded 95% confidence intervals for the predicted values. For shivering, log_10_(frequency) = 1.335–1.86 log_10_(body mass), and for shaking dry, log_10_(frequency) = 1.063–0.215 log_10_(body mass). Circles are data from mammals used to calculate the scaling relationships for shivering and shaking dry. Black triangles are mean frequencies for hummingbirds (triangle pointing left) and seals (triangle pointing down) shaking dry. Orange and purple symbols are mean frequencies for courtship displays of other bird species (note that “S-t grouse” is the sharp-tailed grouse). Data sources are listed in S2 Text.

The average feather-shaking frequencies for peafowl train-rattling and covert-rattling displays are above the predicted values for an animal the mass of a peacock shivering and shaking dry, respectively (Fig 2). On the other hand, peacock wing-shaking and train-shivering frequencies (Table 1) are within the range of predicted values for an animal the mass of a peacock shaking dry (Fig 2).

### 3.3 Train-Rattling Vibration Frequencies

**Fig 3:**
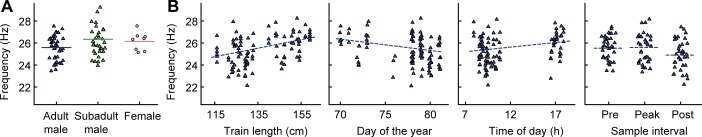
**Peafowl feather vibration frequencies during displays in relation to (A) sex, age, and (B) other factors that predict feather vibration frequencies in adult males.** (A) Displaying peafowl vibrate their feathers at frequencies ranging from ~22–30 Hz, with broad overlap in the ranges of frequencies used by different sex and age classes. Horizontal lines in A are grand means. Dashed lines in B are fits from the linear mixed effects models described in section 3.3.

During train-rattling displays adult peacocks shook their feathers at slightly lower peak vibration frequencies than either subadult males or females performing train- and covert-rattling displays (Fig 3A; Table 1), but the differences were not statistically significant (LMEM, LR chi-square = 3.60, p = 0.17, df = 2; n = 80 displays by 33 individuals).

For adult males, most (60%) of the variation in peak vibration frequency was due to differences among individuals. Adult peacocks with longer trains shook their feathers at slightly higher frequencies on average (Table 2), such that a 43 cm difference in train length (116 to 159 cm) resulted in an increase of only 1.7 Hz [0.5, 2.8] (Fig 3B). A male’s train-rattling vibration frequency decreased slightly with advancing date during the mating season, increased later in the day, and decreased after it reached peak amplitude during a train-rattling bout (Table 2; Fig 3B). In the averaged model, 35% of the total variance in frequency (R^2^_GLMM(m)_) was explained by train length, date, time of day, and sample interval, and half of that explained variance was accounted for by train length alone. The top models (∆AICc < 2) did not include tail length (as opposed to train length), the number of eyespots displayed, or air temperature, so we conclude that none of these variables have an appreciable influence on vibration frequency (S7 Text).

**Table 2:** LMEM to predict peacock train-rattling vibration frequency (*n* = 104 observations of 35 displays by 12 adult males).

	estimate* [95% CI]	standardized beta** [95% CI]
intercept	27.6 [19.6, 35.6]	
sample interval		
before peak interval	–0.07 Hz [–0.40, 0.26]	–0.03 [–0.15, 0.10]
after peak interval	–0.69 Hz [–1.03, –0.36]	–0.25 [–0.37, –0.13]
time of day	0.08 Hz hour^-1^ [–0.01, 0.17]	0.23 [–0.02, 0.49]
day of the year	–0.10 Hz day^-1^ [–0.19, –0.01]	–0.26 [–0.50, –0.02]
train length†	0.04 Hz cm^-1^ [0.01, 0.07]	0.40 [0.12, 0.67]

*Estimates are from the average of the two top models (∆AICc ≤ 2).

**Standardized beta is determined by standardizing predictors and provides a unitless measure of effect size that can be compared among predictor variables.

†Train length is corrected for growth between the date of measurement and the date that the display was recorded (S1 Text).

### 3.4 Audio Components of the Train-Rattling Display

**Fig 4:**
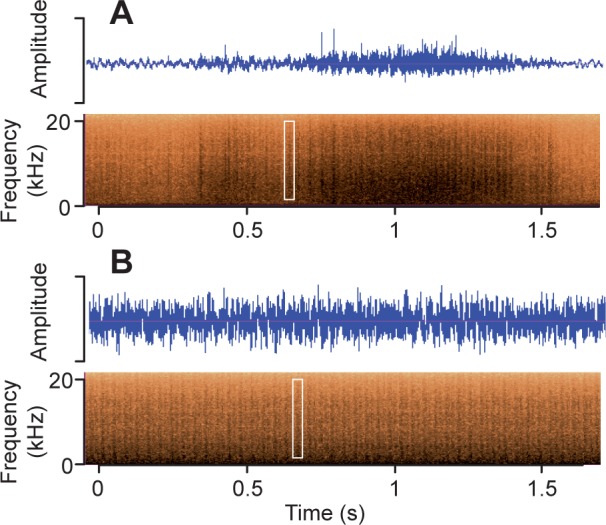
Train-rattling produces a broadband, pulsating sound. Each white box highlights a single rattle note. (A) Relative waveform amplitude (in arbitrary units) and spectrogram of the mechanical sound produced when a peacock performs the train-rattling display. The variation in intensity from 0 to 1 s is due to the peacock modulating the amplitude of his feather vibrations. (B) An array of tail feathers shaken at the train-rattling frequency in the laboratory produced a mechanical sound similar to that shown in A.

The rattle sounds recorded in the field during the train-rattling display consist of short, broadband pulses (Fig 4A) repeated at a rate of 25.6 Hz [24.7, 26.5] (grand mean [95% CI], *n* = 30 recordings of 10 adult peacocks), the same as the average feather vibration frequency measured in the field videos. Two results suggest that the rectrices may produce a substantial component of the train-rattling sound via stridulation. First, audio recordings had the highest amplitude directly behind the tail (S3 Text). Second, audio recordings made when the rectrix array was shaken at 25.6 Hz in the laboratory (Fig 4B) produced spectrograms with broadband frequency ranges and pulse durations that were similar to those of actual train-rattling spectrograms recorded in the field.

### 3.5 Visual Components of the Train-Rattling Display

The eyespots near the center of the train moved during train-rattling with a maximum displacement of 1.7 mm [1.3, 2.1] in the lateral plane, and the eyespots on the longest feathers at the sides of the train had a maximum lateral displacement of 1.1 mm [0.9, 1.3] (grand means [95% CI], *n* = 18 eyespots from 6 males for each location). By contrast, the iridescent loose barbs near those eyespots had a maximum lateral displacement due to train-rattling of 9.6 mm [3.9, 15.3] (*n* = 13 barbs from 5 males). For a peahen in front of the male at a distance of 1 m from the train, the mean lateral displacement of the most mobile eyespots corresponds to a visual angle of about 0.1°, which is equal to the theoretical resolution limit for peafowl (Hart, 2002). Thus, for a peahen located in the typical position during the display, the eyespots would appear to be almost stationary relative to the oscillating loose barbs, even at their peak amplitude of vibration.

The relative lack of eyespot motion is also consistent with the train feathers oscillating in a normal mode pattern with the eyespot very close to a node. To further evaluate whether the feather motion was consistent with normal modes, we used the methods described in section 2.3 to determine the spatial pattern of vibrations for 9 males performing train-shivering and train-rattling at a steady frequency. In each case we determined that the feather motions corresponded to normal modes. When peacocks performed train-shivering behavior (S3 Movie), the rectrices oscillated in a pattern consistent with the *k* = 1 normal mode for all but a few of the longest feathers, which oscillated in the *k* = 2 mode, whereas the longest train feathers oscillated in either the *k* = 3 or 4 normal modes (e.g., Fig 1F). During train-rattling, the shortest, medium, and longest rectrices oscillated in a pattern consistent with the *k* = 1, 2, and 3 normal modes, respectively (Fig 1E). Although it was difficult to obtain images of the rachis motion of train feathers during train-rattling (because the peacocks also tilted and pivoted their trains), we were able to track the motion of antinodes and determined that train feathers also oscillated in normal modes during train-rattling.

### 3.6 Feather Vibrational Resonance

When single rectrices were shaken in the laboratory at 25.6 Hz, they oscillated in the same modes as those observed during train-rattling in the field. When single eyespot feathers were shaken at their *k* = 1 normal mode frequency, the eyespots moved freely, but when they were shaken at 25.6 Hz, the eyespots barely moved (≤ 6–9% of eyespot diameter; S4 Movie) because they were always located immediately distal to a node.

**Fig 5:**
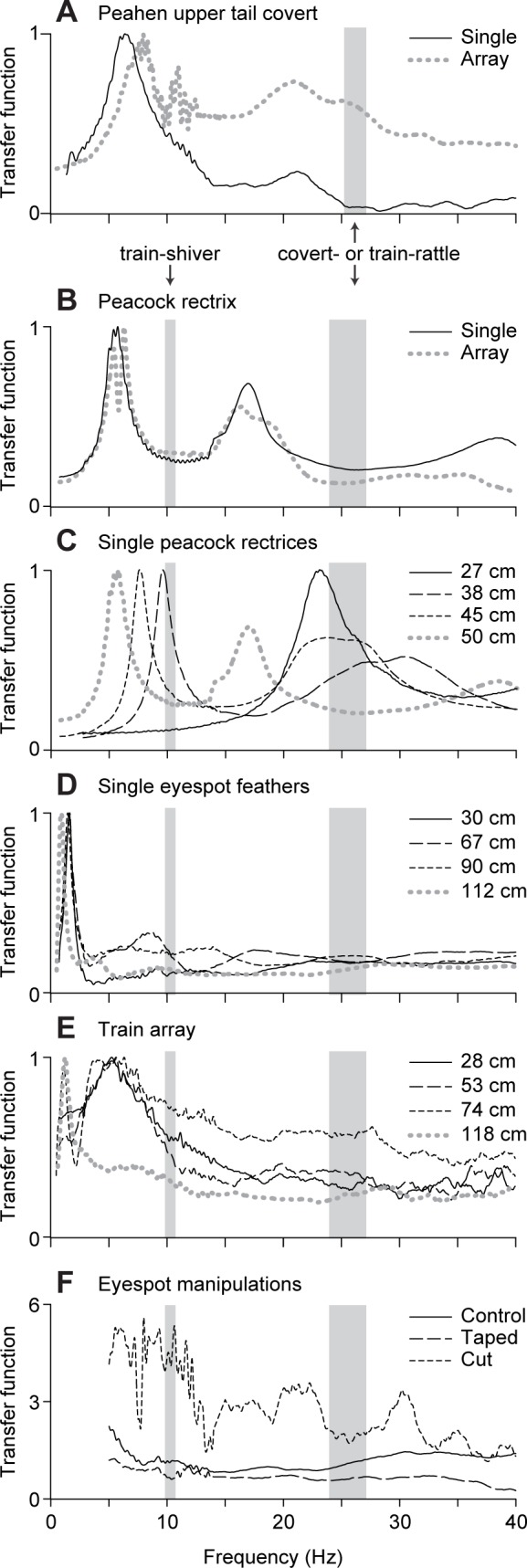
Feather vibrational responses calculated from laboratory feather shaking experiments. Vibrational spectra are plotted as drive transfer functions (i.e., the ratio of feather to shaker Fourier magnitudes) vs frequency. Gray shaded regions indicate the range of individual mean shaking frequencies for peahen covert-rattling (25.2–27.1 Hz) in A and adult peacock train-shivering (9.8–10.7 Hz) and train-rattling (23.9–27.1 Hz) in B-F (see Table 1 for details). Sample spectra are shown for (A) one peahen tail covert feather and an array of peahen tail coverts, (B) one peacock rectrix and an array of peacock rectrices, (C) four peacock rectrices of different rachis lengths (*L*_*R*_) shaken singly, (D) four eyespot feathers of different rachis lengths shaken singly, (E) four eyespot feathers of different rachis lengths shaken in an array of train feathers. (F) Vibrational spectra measured to determine the effect of altering the mass at the distal top of an eyespot feather: a single eyespot feather (*L*_*R*_ = 111 cm) unaltered (Control), with added mass (Taped), and with the eyespot removed (Cut). Note that in F data were omitted below 5 Hz to emphasize changes for frequencies close to peacock shaking behaviors. All lengths are the rachis length, *L*_*R*_.

Examples of resonant spectra are shown in Fig 5. Spectral peaks for single feathers were well approximated by the Lorentzian functional forms predicted for elongated structures oscillating in a single plane, in agreement with our observation that the shaken feathers show minimal torsion and little movement out of the lateral plane.

For the eyespot feathers, the *k* = 1 to 4 normal modes had mean *Q*_*k*_ ranging from 3.6–4.5 (± 0.4 SE), whereas *Q*_*k*_ ≥ 5 for nearly all rectrices and the mean ± SE for the first three modes were *Q*_*1*_ = 7.8 ± 0.5, *Q*_*2*_ = 5.6 ± 0.7, and *Q*_*3*_ = 4.9 ± 0.4 (S5 Text). These values imply that each tail and eyespot feather has relatively broad resonant peaks due to air drag and internal energy dissipation.

On live birds, feathers are not vibrated in isolation. To understand how interactions among feathers might influence their resonant properties, we performed similar measurements on feather arrays. For the rectrix array and the female tail covert array, the resonant frequencies of the *k* = 1 and 2 normal modes were similar to those of single feathers of the same length and type. Each resonant peak in the single feather spectrum was replaced by a slightly wider compound peak in the corresponding feather array spectrum (Fig 5A and 5B). Thus, interactions between the feathers in these arrays were weak [21] and primarily resulted in a slightly reduced *Q*_*k*_. Higher order vibrational modes of the rectrix array were also substantially damped. In contrast, feathers in the train array had broad spectra that were very different from those of individual eyespot feathers (Fig 5D and 5E), with *Q*_*k*_ greatly reduced (e.g., mean *Q* ± SE: *Q*_*1*_ = 1.8 ± 0.3, *Q*_*2*_ = 1.5 ± 0.5, *Q*_*3*_ = 2.7 ± 0.6). All but the shortest feathers in the train array had the same peaks in their vibrational spectra: a *k* = 1 peak at 1.2 Hz, a broad *k* = 2 peak centered at ~6 Hz that extended into the frequency range of train-shivering (10.4 Hz), and a broad peak centered at ~20–30 Hz that includes the train-rattling frequency (25.6 Hz). The lack of length-dependence for resonant peaks in the train array indicates that the interactions among train feathers coordinate their motion such that they respond as a unit.

Fig 5F shows that adding mass at the end of a long eyespot feather reduced its motion but did not affect its resonant frequencies. Conversely, cutting off the eyespot increased the vibrational amplitude of the tip of the altered feather, instead of decreasing it as predicted by cantilever theory when the length of a beam is reduced. These results suggest that the limited motion of the eyespot is due to its mass and the compact structure of its interlocking barbules, and not only the properties of the feather rachis.

**Fig 6:**
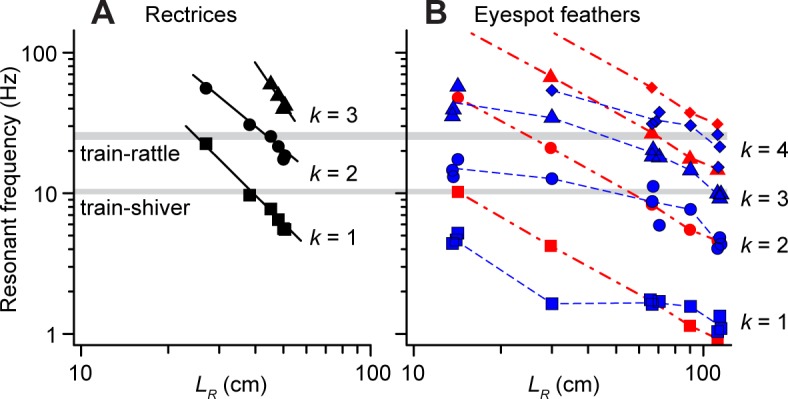
Resonant frequency of single peacock feathers as a function of length. Values of resonant frequency *f*_*k*_ for several normal modes of vibration measured in the laboratory for (A) rectrices (black symbols, solid lines) and (B) eyespot feathers (blue symbols, dashed lines). Horizontal gray bands indicate the range of mean frequencies for train-rattling displays (grand mean = 25.6 Hz) and train-shivering behavior (grand mean = 10.4 Hz) performed by adult males (see Table 1 for details). Solid black lines in A show power law scaling relationships for the rectrices calculated from the resonant frequency data. In B the blue dashed lines connect the mean values for each of the five eyespot feather lengths studied. Note that the resonant frequencies of the eyespot feathers in B have a much weaker length-dependence than the rectrices in A. Red symbols and dot-dashed lines in B show the theoretical predictions for the eyespot feathers as described in the text (Eq 3).

Fig 6 shows how each resonant frequency, *f*_*k*_, of a feather scales with its rachis length for both rectrices and eyespot feathers. For rectrices, the relation between *f*_*k*_ and *L*_*R*_ agreed with a power law (*f*_*k*_ ∝ *L*_*R*_−^*b*^) where *b* = 2.2 [1.9, 2.5], 1.7 [1.5, 1.9], and 3.0 [2.8, 3.2] for *k* = 1, 2, and 3, respectively (Fig 6A; *R*^2^ > 0.99, 0.98, and 0.99; *n* = 8, 8, and 4 feathers, respectively). This indicates that the frequencies for the *k* = 1 and 2 normal modes scale with rectrix length as predicted for uniform cantilevers (i.e., *b* = 2) [21]. By contrast, for eyespot feathers, each resonant frequency, *f*_*k*_, depended only weakly on feather length (Fig 6B). We therefore developed a theoretical model to explain this last result, as described in section 3.5 below.

Fig 6 also compares the shaking frequencies from our field data with the resonant frequencies of individual peacock feathers with different lengths, *L*_*R*_. For a given feather length and vibration frequency, the closest resonant frequency *f*_*k*_ should determine which normal mode pattern (*k*) is excited by the vibration, assuming that the interactions between the feathers are weak. From Fig 6A, we see that the train-rattling frequency is closest to the resonant frequency for the *k* = 1, 2 and 3 modes of the shortest, medium and longest rectrices, respectively. This is indeed the pattern of normal modes that we observed for those rectrices in the field videos (section 3.5, Fig 1E). Similarly, the frequency of train-shivering in Fig 6A is closest to the *k* = 1 resonant frequencies for most rectrices, except for the longest rectrices which should oscillate in the *k* = 2 normal mode. Again, this is consistent with our observations from the field videos. Given that interactions among the train feathers had a substantial effect on their vibrational responses at high frequencies (Fig 5E), we did not evaluate the normal modes of train feathers during the train-rattling display. However, during train-shivering, when the train feathers are much more widely spaced, eyespot feathers with *L*_*R*_ ~100 cm should oscillate in the *k* = 3 normal mode (Fig 6B). Extrapolating from our laboratory data, longer eyespot feathers should oscillate in the *k* = 3 or 4 mode, and this is what we observed (Fig 1F).

**Fig 7:**
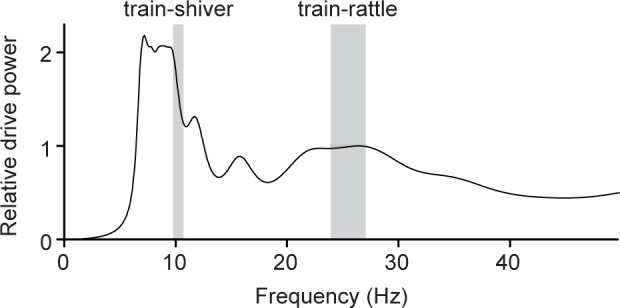
Predicted power spectrum for a peacock’s tail as a function of the vibrational frequency. These values were computed from laboratory data and the distribution of feather lengths in an individual peacock’s tail, using the mathematical model described in S8 Text. Note that “tail” refers to the array of rectrices, not the elaborate train. This model predicts that the tail has two broad resonant peaks near the average train-shivering and train-rattling frequencies that also agree with resonant peaks in the vibrational spectral response of the train array (Fig 5E). Gray shaded regions indicate the range of individual mean frequencies for shaking behaviors (Table 1).

Our results indicate that peacock rectrices interact only weakly with each other, based on their similar vibrational spectra and normal modes when shaken either singly, in the rectrix array, and during natural train-rattling displays. This allowed us to create a mathematical model of the drive power transferred from the peacock’s muscles to its tail, based on tail morphology and rectrix vibrational spectra (S8 Text). Fig 7 shows one example of the resulting power spectrum predicted by this model for the morphology of the tail of a particular peacock (note other individual tail morphologies gave similar model spectra; *n* = 12 model power spectra, each based tail morphology data from a different peacock). These model spectra each have a broad resonant peak centered at 21.9 Hz [20.2, 23.6], on average, that overlaps with the average vibration frequency of feathers during train-rattling (Fig 7), and that also agrees with the broad *k* = 3 spectral peak for the train array at ~27 Hz (Fig 5E). The breadth of this peak in the model suggests that peacocks can efficiently excite their tails to drive the train vibrations at a range of frequencies from 22–27 Hz (Fig 7). Each model spectrum has another major peak centered at 6.9 Hz [6.2, 7.6], on average, that is close to both the average train-shivering frequency (Fig 7) and the *k* = 2 spectral peak for the train array (Fig 5E). These results indicate that the tail should be able to efficiently drive train vibrations at both the train-rattling and train-shivering frequencies. The first and second peak frequencies in each model spectrum decreased with increasing tail length (slopes of –0.32 Hz cm^-1^ [–0.39, –0.25] and –1.0 Hz cm^-1^ [–1.4, –0.6], respectively). Finally, none of the model spectra for the tail had a peak below ~5 Hz (Fig 7), which may explain why we do not see the *k* = 1 mode excited in the peacock’s train in our field videos (because the train array has its *k* = 1 peak at about 1.3–1.6 Hz; Fig 5E).

### 3.7 Eyespot Feather Biomechanical Properties

**Fig 8:**
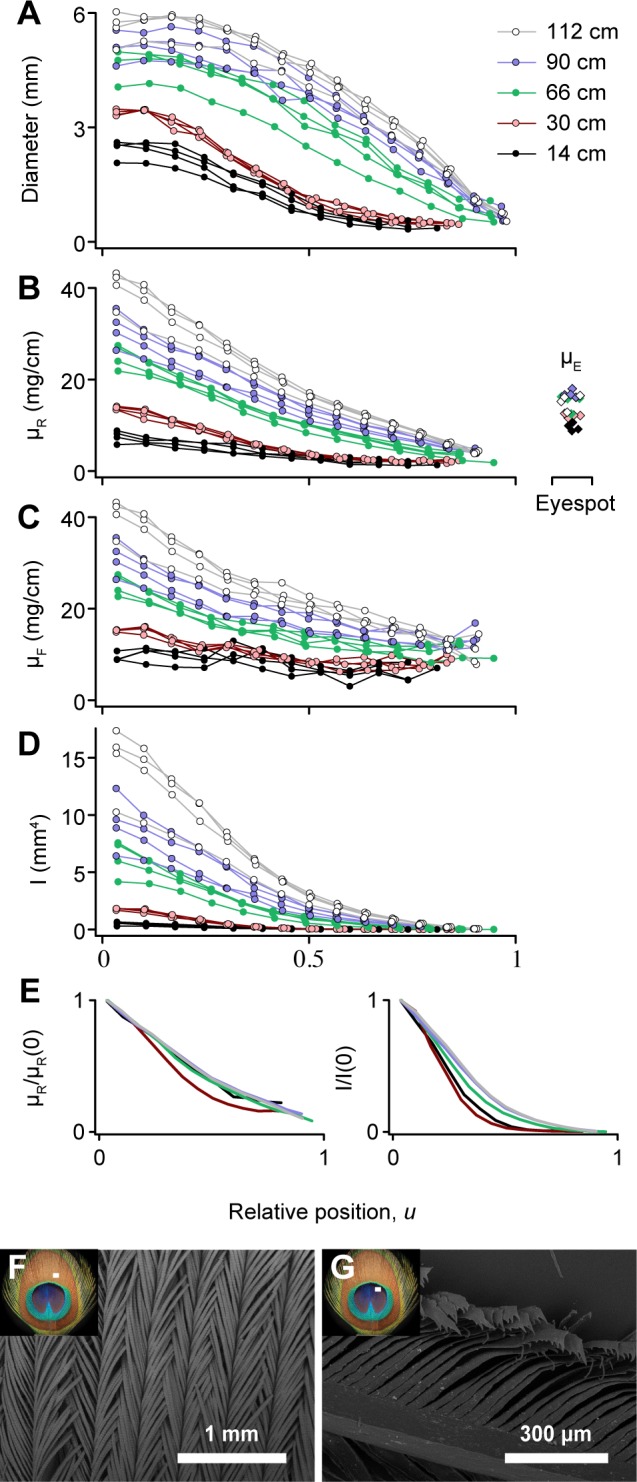
Morphometrics, mechanical parameters, and ultrastructure of eyespot feathers. Plots A-D show how feather morphology (*n* = 4 feathers of each length) varies with position along the rachis, *x*, rescaled to be a fraction of rachis length, *u* = *x*/*L*_*R*_: (A) rachis diameter, *D*_*1*_, in the lateral plane (parallel to the barbs), (B) linear density of the rachis, *μ*_*R*_, (C) linear density of the entire feather, *μ*_*F*_, and (D) second moment of area for the rachis in the lateral direction, *I*, computed from the diameter and linear density data. Linear density of the eyespots, *μ*_*E*_, is shown to the right of the rachis data in B. To compare the functional forms for *μ*_*R*_ and *I* for different length feathers, the plots in E show the average values of *I* and *μ*_*R*_ rescaled for each feather to have a value of one at *u* = 0. SEM images show that peacock eyespots have (F) intermeshed barbules and (G) microhooks that help the eyespot move as a single unit. Insets indicate the approximate locations of the barbules shown in the SEM images with white rectangles.

Fig 8A and 8B shows the rachis diameters and linear density profiles for different eyespot feathers at relative positions along their lengths, *u* = *x*/*L*_*R*_. From these data we computed the second moment of area, *I*, of eyespot feathers in the lateral plane where feather vibrations occur during train-rattling (Fig 8D). Although the rachises of longer eyespot feathers are stiffer and denser than those of shorter eyespot feathers, they have similar functional forms for all feather lengths when the linear density profiles and the second moment of area of the rachis are scaled to have a value of one at *u* = 0 (Fig 8E). This is surprising because the shape of the rachis in eyespot feathers varies with feather length such that *L*_*R*_/*D*_*avg*_ ranges from 95 (*L*_*R*_ = 14 cm) to 293 (*L*_*R*_ = 112 cm), where *D*_*avg*_ is the mean diameter of the rachis. This indicates that variation in the internal structure of the rachis must compensate for the variation in its shape along its length.

Fig 8B also shows the linear densities of the eyespots in comparison to the part of the rachis that supports the eyespots. The eyespot is denser than the proximal parts of the feather for all but the shortest feathers. Furthermore, SEM images of the eyespot revealed that the barbules are densely intermeshed and have microhooks along their edges (Fig 8F–8G), thus explaining how the eyespot can move as a single mass. By contrast, the nearby loose barbs are widely spaced and free to move as independent cantilevers.

To understand how all these factors influence the length-dependence of the resonant frequencies of eyespot feathers in Eq 1, we computed the average of the ratio of the second moment of area in the lateral direction to the linear feather density, *(I*/*μ*_*F*_)_*avg*_, for each eyespot feather. We approximated *G´* as 3.3 GPa, the longitudinal Young’s modulus for the cortex of an eyespot feather [35], and the effect of the eyespot mass, *M*_*E*_, at the tip using

$$f_{k}\approx K_{k}^{2}\frac{1}{2\pi L_{R}{}^{2}}\sqrt{\frac{G^{\prime }{\left(I/\mu_{F}\right)}_{avg}}{\left(1+4.1\frac{M_{E}}{M_{F}}\right)}}$$ (3)

where *M*_*F*_ is the mass of the rest of the feather [36]. We used *K*_*1*_ = 1.875, the value for a uniform cantilever with a freely-moving tip, to model eyespot feathers oscillating in the *k* = 1 mode; for our values of *Q*, the resonant frequency shifts ≤ 2% [29]. For higher order normal modes, we used *K*_*k*_ for a uniform cantilever with a hinged-tip (where *K*_*k*_ = 3.927, 7.069, and 10.21 for *k* = 2, 3, and 4, respectively) because there is a node just below the eyespot. The scaling with length predicted by Eq 3 (Fig 6B) agrees well with that of measured resonant frequencies for eyespot feathers with *L*_*R*_ ≥ 66 to 80 cm. Eq 3 overestimates resonant frequencies for longer feathers (*L*_*R*_ ≥ 80 cm) by ≤ 21% for *k* = 1 and 2 and by ≤ 64% for *k* = 3 and 4, whereas it overestimates the resonant frequency of all normal modes of shorter feathers by ≤ 280%.

Several factors may contribute to these discrepancies. First, we did not solve the differential equation for frequency because a more exact treatment would require modeling the many long, loose barbs that function as coupled oscillators that interact with the rachis. Second, the elastic modulus for long eyespot feathers exceeds that predicted by their individual components due to reinforcing interactions between the cortex and pith [30]. We also estimated that the volume of air displaced by the feather vibrating in the lateral plane is less than or equal to that of the nearby feather [37]. This implies that the ratio of added air mass to feather mass is less than or equal to the ratio of their densities. Given that the density of air is < 1% of that of the entire feather, including the air gaps between the loose barbs, we conclude that the effect of the added air mass is negligible. Finally, our model did not account for potential variation in elastic modulus along the rachis [38].

The computed values of critical stress for local buckling were greater than those for Euler buckling along almost the entire length of the rachis for all eyespot feathers, with the lowest values near the proximal end. This level of critical stress ensures that the eyespot feathers bend appreciably before developing unrecoverable kinks. However, too much elastic bending would make it impossible to hold the train erect during displays, so we would expect greater bending stiffness (hence larger *I*) in the dorsoventral than in the lateral direction. We indeed found that the dorsoventral value of *I* near the base of the feather was greater than or equal to the lateral value, whereas at the distal end the dorsoventral *I* was three to nine times the lateral value. Thus, the eyespot feathers are stiffer front-to-back than sideways, consistent with train-rattling vibrations occurring only laterally.

## 4. Discussion

Courting peacocks perform the train-rattling display using a relatively narrow range of feather vibration frequencies between 22 and 28 Hz (Fig 3), and yet within that range we found that different individuals use relatively consistent vibration frequencies. What determines one peacock’s ability to perform higher frequency vibrations than another? We analyzed data from a limited number of individuals to investigate whether variation in train morphology constrains vibrational frequency, and found that adult peacocks with longer trains shook their trains at slightly higher frequencies on average (Fig 3B). This indicates that having a larger train does not constrain peacocks to perform vibrations at lower frequencies. Given that train mass is highly correlated with train length [24], our results also suggest the possibility that longer-trained males may perform displays that require more vibrational power. Although we cannot address this hypothesis with our data, it could be tested by measuring metabolic power requirements of displays performed by individuals with trains of different lengths. Although natural variation in train length is not associated with peacock mating success [10], it is positively correlated with fat reserves [24], suggesting that increased energetic reserves of longer-trained males may allow them to perform more costly displays. Train length is also associated with circulating androgen levels [39], which may influence a peacock’s energetic reserves, his motivation, or his capacity for muscular force production that is independent of other factors. The muscles used to spread and fan the train and tail during displays are also sexually dimorphic [40], but the extent of variation among males is not yet known. Further study is needed to evaluate whether the frequency and amplitude of feather shaking are modulated depending on social factors such as the presence of females or competing males. To evaluate individual metabolic capacity and muscle power output, experiments could be performed where mass is added to the train to examine how this influences display vibration frequency, duration, and the metabolic costs of displays performed by different males.

We also found that the range of frequencies used by peacocks performing the train-rattling display lies well above the scaling relationships with body mass for other types of shaking behaviors (Fig 2). This is not true of all avian courtship displays: e.g., the frequencies of wing shaking by peacocks and other birds and tail-clicking by sharp-tailed grouse lie near or below the values predicted for animals of that size shaking themselves dry; the same is true for train-shivering behavior performed by adult peacocks (Fig 2).

Our results point to several biomechanical features of the peacock’s tail and train feathers that allow them to vibrate at such high frequencies during the train-rattling display. First, we show that the frequencies used during train-rattling are well-matched to a peak in the resonant response of the peacock’s tail (Fig 7). This means that displaying peacocks shake their tails at or near resonance when they excite train vibrations during this display. Second, we found that arrays of peacock eyespot feathers are also in resonance at the train-rattling frequency. This occurs because the resonant frequencies of these feathers depend only weakly on feather length (Fig 6B). When multiple eyespot feathers of different lengths are combined into an array, as they are in the peacock’s train, this weak length-dependence promotes their ability to oscillate at similar resonant frequencies (Fig 5E). This agreement in resonant frequency between the tail and the array of train feathers reduces the power required to drive vibrations.

A third feature that facilitates the peacock’s display is the relatively high damping of the train and tail feathers when they are shaken at frequencies and amplitudes typical of the train-rattling display. Eyespot feathers have *Q* ≤ 6 at the train-rattling vibration frequency, whereas the feathers that manakins (family Pipridae) use to sonate have *Q* > 10 [27]. The higher damping of peacock feathers broadens the resonant peaks of their train and tail, helping to explain how a single vibrational frequency can be used to move such a large array of feathers relatively efficiently. This also has the advantage of quickly damping out oscillations caused by external sources (e.g., wind).

Although the rachis of an eyespot feather is superficially similar to other keratinized structures, including mammalian whiskers and quills, its shape and composition appear to be well-suited to a unique role. Whiskers are truly conical, providing them with extra flexibility and resonant frequencies that are strongly dependent on whisker length, both of which support their sensory function [25, 41]. Porcupine quills are nearly cylindrical and stiff enough to hold erect and rattle during threat displays [42]. By contrast, the composition and shape of the peacock’s eyespot feather rachis facilitates driving these long feathers to vibrate at high frequencies, while allowing them to remain erect during displays and to support the relatively heavy eyespots without undue bending. When peacocks hold their trains erect, they risk damage to their train feathers caused by wind, assaults by other males, and other causes. Thus, it is not surprising that the mechanical design of their train feathers protects against local buckling, which is not recoverable, at the expense of appreciable elastic bending.

The position dependence of bending stiffness in the lateral direction for the eyespot feathers (as shown by *I* in Fig 8D) is similar to that found for flight and tail feathers in other bird species [38, 43]. However, the magnitude of the second moment of area, *I*, for short eyespot feathers is only 28% of that of owl and pigeon flight feathers of similar length [38]. This greater flexibility of the eyespot feathers also facilitates vibrations at large amplitudes during displays.

It is intriguing that the colorful eyespots–which influence peahen mate preferences [12]–remain so steady during the peacock’s train-rattling display (S1 Movie). The low amplitude of eyespot motion is not solely the result of interactions between feathers, because their motion was similar even when feathers were shaken individually at 20–30 Hz in the laboratory (S4 Movie). SEM imaging also revealed barbule ultrastructures that lock together the eyespot barbs (Fig 8F and 8G), similar to those found on flight primaries and contour feathers in other bird species [44]. Thus, each eyespot moves as a single unit with relatively greater mass than the surrounding loose barbs, and experiences only a weak restoring force from the tapered distal end of the rachis (Fig 8A, 8B and 8D). In agreement with this model, we found that adding mass to the eyespot reduced its range of motion, whereas removing the eyespot greatly increased the amplitude of tip motion (Fig 5F).

Given that train-rattling always precedes copulation, and attracts a peahen’s gaze [15], is it possible that the dynamics of this display influence female mate choice? The available evidence indicates that peahens are highly sensitive to both the visual stimuli and the sounds produced by train-rattling. For example, peahens should be able to visually resolve the iridescence of the loose barbs moving at 20–30 Hz, given that all bird species studied to date can resolve flicker at much higher rates [45]. The rapid motion of the loose barbs at this frequency of feather shaking creates a dynamic iridescent background around each eyespot (S1 Movie). It is possible that this motion also influences how peahens perceive the eyespot colors that are important for mate choice [11, 12, 46, 47].

In addition, the frequency range of the broadband rattle notes produced by train-rattling lies within the range of peafowl auditory response curves predicted from their inner ear morphology [48]. Peafowl have been shown to perceive playbacks of even the very low frequency (≤ 20 Hz) components of train-rattling [13]. This is not unique among birds as parakeets are sensitive to amplitude modulations of broadband noise at 20–30 Hz [49]. Although our study does not address whether females are attracted to males that produce particular feather sounds, future studies could use playback experiments to address this question.

One hypothesis for future studies is that the dynamics of these high frequency train vibrations may signal a peacock’s capacity for muscle power output [7, 8]. When a peacock displays, it moves the mass of the train (0.3 kg; [24]) as well as that of supporting muscular, connective, and dermal tissue. We often video-recorded peacocks train-rattling for > 25 minutes, suggesting that this behavior may pose a considerable challenge to metabolic stores and short-term muscle power output. Assuming sound intensity is proportional to the power required to drive feather vibrations, the acoustic intensity of this multimodal display may allow females to evaluate the power output of different males.

In his book on sexual selection, Darwin noted that “Peacocks and Birds of Paradise rattle their quills together, and the vibratory movement apparently serves merely to make noise, for it can hardly add to the beauty of their plumage” [vol. 2, page 61] [50]. On the contrary, our results suggest the possibility that sexual selection (via female choice) has shaped both the biomechanical design of the eyespot feathers and the behaviors that produce visual and audio cues. The peacock’s display is influenced by the location and structure of both the eyespots and the loose barbs that surround them, the shape of the eyespot feather rachises, and the frequency at which those feathers are vibrated during displays. Further work is needed to determine whether females use variation in those audiovisual features to discriminate among males, and what benefits they might gain by such discrimination–or whether Darwin was right and it is all just noise.

## Supporting Information

S1 Datasets Datasets and R script. (ZIP)

S1 Movie Real-time and slow motion video of peacock courtship displays. https://www.youtube.com/watch?v=voPTJ9KKsoY Adult peacocks court peahens by vibrating the erect tail and elongated upper tail covert feathers during their train-rattling displays. Wing-shaking is also performed during courtship. Slow-motion clips of train-rattling peacocks demonstrate that the eyespots remain relatively stationary (as compared to the rest of the eyespot feather), and that the tail drives the vibrations by stridulating against the train feathers.
(MP4)

S2 Movie Subadult peacock train-rattling and peahen covert-rattling displays. https://www.youtube.com/watch?v=nCaZ0wYpJPc Subadult peacocks (i.e., those with trains < 1 meter in length) also perform train-rattling displays. Peahens perform a similar behavior that we call covert-rattling, in which they vibrate their tails and upper tail coverts.
(MP4)

S3 Movie Peacocks perform train-shivering to erect and spread their train feathers. https://www.youtube.com/watch?v=zatLFC5wVn0 Videos are shown from the front and side, in real-time and slow motion.
(MP4)

S4 Movie Real-time and high-speed video of a peacock eyespot feather vibrated by a mechanical shaker. https://www.youtube.com/watch?v=yTfk5Izepfo When peacock eyespot feathers are vibrated at 25.6 Hz using a mechanical shaker, the eyespot remains relatively stationary compared to the rapidly moving and flickering loose green barbs. This motion mimics what is observed during natural courtship displays. The 25.6 Hz frequency used in this example is the average frequency of peacock train-rattling displays.
(MP4)

S1 Text Growth of peacock feathers. (DOCX)

S2 Text Sources of shaking frequencies and scaling relationships used in Fig 2 of the main text. (DOCX)

S3 Text Audio analysis and characterization of sound production. (DOCX)

S4 Text Calculation of mechanical properties of the feather rachis. (DOCX)

S5 Text Feather vibrational resonance. (DOCX)

S6 Text Comparison of eyespot motion and peafowl visual acuity. (DOCX)

S7 Text Statistical models of morphology and train-rattling frequency. (DOCX)

S8 Text Model of resonant properties of the tail. (DOCX)

S9 Text Statistical supplement. (PDF)

## Abbreviations

L: feather length
L_R_: length of feather rachis outside the body (i.e., omitting the calamus)
k: normal mode index
f_k_: resonant frequency of the *k*^th^ normal mode
μ_F_: feather linear density (mass per unit length)
G’: dynamic storage modulus
I: second moment of area
K_k_: dimensionless coefficient in the equation for resonant frequency
μ_R_: feather rachis linear density (mass per unit length)
A: FFT magnitude of feather vibrational spectrum at a given frequency
A_d_: FFT magnitude of shaker vibrational spectrum at a given frequency
f_d_: vibrational drive frequency of the mechanical shaker
x: position along the rachis from the feather insertion point
u: position relative to the feather insertion point rescaled to be a fraction of rachis length (i.e., *x*/*L*_*R*_)
D: diameter of the feather rachis
Q_k_: quality factor of the *k*^th^ normal mode
Δf_k,3dB_: width of the vibrational spectral peak about the resonant frequency *f*_*k*_, such that the width is measured to the points at which the power falls to half its peak value
M_E_: eyespot mass
M_F_: feather mass
b: scaling exponent
