# Supplementary material for: Biomechanics of the Peacock’s Display: How Feather Structure and Resonance Influence Multimodal Signaling
Source: PLoS One. 2016 Apr 27;11(4):e0152759. doi: 10.1371/journal.pone.0152759 (PMC4847759; doi:10.1371/journal.pone.0152759)
Supplement: S1 Text — (DOCX) [file pone.0152759.s006.docx]

**S1 Text. Growth of peacock feathers**

Because the peacock’s train is still growing during the breeding season, we corrected our measures of train length to obtain an estimate on the date of a recorded display. We first fit a three-parameter logistic growth curve to data pooled from [1] and the present study (Fig S1). Over the range of dates of displays video-recorded in this study (11-22 March), this curve is closely approximated by a straight line (Fig S1). Hence, to compute a growth rate for the dates of interest in this study (11-22 March), we used the slope of the linear approximation of the fitted growth curve over those dates, and we determined the 95% confidence interval for this estimate by bootstrapping the three-parameter logistic fit (10,000 replicates). This yielded a growth rate of 0.41 cm day^-1^ [0.31, 0.51 cm day^-1^] over the period of our study. Fits to different types of growth curves yielded similar estimates for the average growth rate over this period (Gompertz: 0.39 cm day^-1^; lognormal: 0.43 cm day^-1^) and using these values resulted in no change in conclusions from our analyses.

The average tail length in this study (49.5 cm [47.7, 51.3]) in March was similar to that noted in [2] for peacocks caught in the UK from October to April (54.0 cm [49.5, 58.5]), suggesting that, unlike the elongated train, the tail is not still growing when the males are courting females.

**
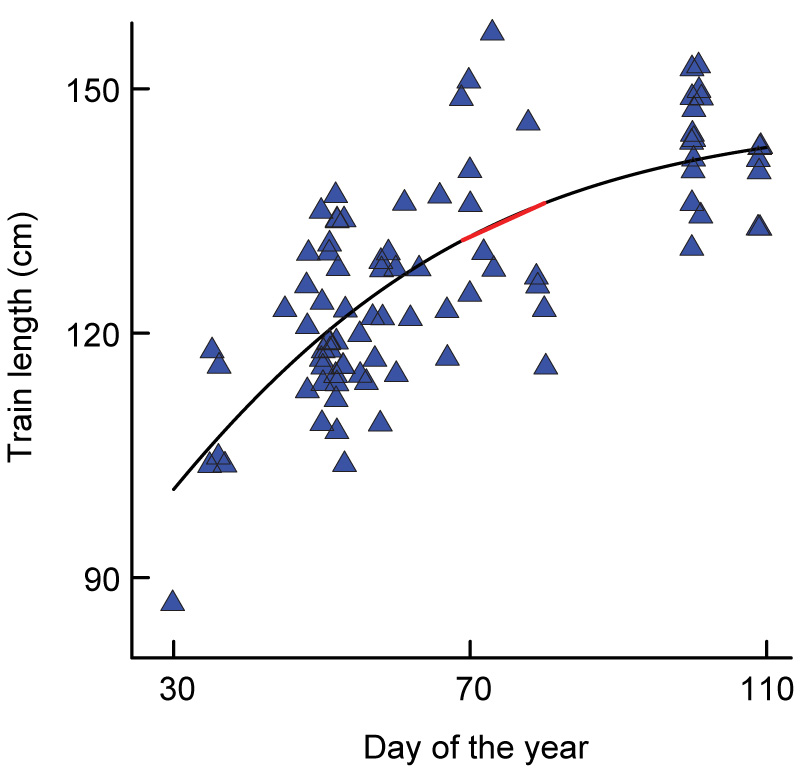
**

**Fig S1. Adult male peacock train length vs day of year.** Train feathers of adult peacocks grow continuously throughout the mating season from March-May, with considerable variation in train length among individuals. The black line represents the three-parameter logistic growth curve, and the red line segment represents its linear approximation over the period when courtship displays were recorded in this study. Because the linear approximation so closely matches the fitted curve over the period of this study, we used its slope when correcting for train growth rate per day in this study.

**References**

1. Dakin R, Montgomerie R. Peahens prefer peacocks displaying more eyespots, but rarely. Animal Behaviour. 2011;82(1):21-8. doi: 10.1016/j.anbehav.2011.03.016. PubMed PMID: WOS:000291476500002.

2. Petrie M, Halliday T, Sanders C. Peahens prefer peacocks with elaborate trains. Animal Behaviour. 1991;41:323-31. doi: 10.1016/s0003-3472(05)80484-1. PubMed PMID: WOS:A1991EZ05800015.
