# Supplementary material for: Biomechanics of the Peacock’s Display: How Feather Structure and Resonance Influence Multimodal Signaling
Source: PLoS One. 2016 Apr 27;11(4):e0152759. doi: 10.1371/journal.pone.0152759 (PMC4847759; doi:10.1371/journal.pone.0152759)
Supplement: S2 Text — (DOCX) [file pone.0152759.s007.docx]

**S2 Text. Sources of shaking frequencies and scaling relationships used in Fig 2 of the main text**

| behaviour | class | species | references |
| --- | --- | --- | --- |
| shaking dry | mammals | 16 mammal species and 5 dog breeds | [1] |
| shaking dry | mammals | Northern fur seal | [2] |
| shaking dry | birds | Anna’s hummingbird | [3] |
| shivering for thermogenesis | mammals | 13 mammal species | [4] |
| display (wing-snapping) | birds | 3 manakin species | [5] |
| display (wing-clapping) | birds | flappet lark | [6, 7] |
| display (wing beating) | birds | ruffed grouse | [8] |
| display (tail-clicking) | birds | sharp-tailed grouse | [9] |
| display (wing-rattling) | birds | boat-tailed grackle | [10], [11] audio [12, 13] |
| display (wing-clapping) | birds | ocellated turkey | [14] audio [15, 16] |

**References**

1. Dickerson AK, Mills ZG, Hu DL. Wet mammals shake at tuned frequencies to dry. Journal of the Royal Society Interface. 2012;9(77):3208-18. doi: 10.1098/rsif.2012.0429. PubMed PMID: WOS:000310573100004.

2. Iwata T, Yonezaki S, Kohyama K, Mitani Y. Detection of grooming behaviours with an acceleration data logger in a captive northern fur seal. Aquatic Mammals. 2013;39(378-384).

3. Ortega-Jimenez VM, Dudley R. Aerial shaking performance of wet Anna's hummingbirds. Journal of the Royal Society Interface. 2012;9(70):1093-9. doi: 10.1098/rsif.2011.0608. PubMed PMID: WOS:000302134300027.

4. Kleinebeckel D, Klugmann FW. Shivering. In: Schoenbaum E, Lomax P, editors. Thermoregulation: physiology and biochemistry. New York, NY USA: Pergamon Press; 1990. p. 235-53.

5. Bostwick KS, Prum RO. High-speed video analysis of wing-snapping in two manakin clades (Pipridae: Aves). Journal of Experimental Biology. 2003;206(20):3693-706. doi: 10.1242/jeb.00598. PubMed PMID: WOS:000187393500020.

6. Norberg RA. The flappet lark *Mirafra rufocinnamomea* doubles its wingbeat rate to 24 Hz in wing-clap display flight--a sexually selected feat. Journal of Experimental Biology. 1991;159:515-23. PubMed PMID: WOS:A1991GD87000031.

7. Ryan PG, Marshall HK. Variation in the wing-clapping display of Clapper Larks. Ostrich. 2005;76(1-2):73-7. doi: 10.2989/00306520509485475. PubMed PMID: WOS:000229121600010.

8. Garcia M, Charrier I, Rendall D, Iwaniuk AN. Temporal and spectral analyses reveal individual variation in a non-vocal acoustic display: the drumming display of the ruffed grouse (*Bonasa umbellus, L.*). Ethology. 2012;118(3):292-301. doi: 10.1111/j.1439-0310.2011.02011.x. PubMed PMID: WOS:000300502300011.

9. Gratson MW. Sexual selection for increased male courtship and acoustic signals and against large male size at sharp-tailed grouse leks. Evolution. 1993;47(2):691-6. doi: 10.2307/2410083. PubMed PMID: WOS:A1993KZ71200028.

10. Post W, Poston JP, Bancroft GT. Boat-tailed Grackle (*Quiscalus major*) Ithaca, NY, USA: Cornell Lab of Ornithology; 2014 [cited 2015 August 30]. Available from: <http://bna.birds.cornell.edu/bna/species/207/articles/introduction>.

11. McIlhenny EA. Life history of the Boat-Tailed Grackle in Louisiana. The Auk. 1937;54(3):274-95.

12. Little RS, Kimball JW. ML12643: Boat-tailed grackle: *Quiscalus major*. Ithaca, NY, USA: Macauley Library, Cornell Lab of Ornithology; 1962 [cited 2015 August 30]. Available from: https://macaulaylibrary.org/audio/12643.

13. Keller GA. ML12643: Boat-tailed grackle: *Quiscalus major*. Ithaca, NY, USA: Macauley Library, Cornell Lab of Ornithology; 1992 [cited 2015 August 30]. Available from: https://macaulaylibrary.org/audio/73901.

14. McRoberts JT, T. Rich, C. Rodríguez-Flores, C. Soberanes-González, and M.C. Arizmendi. Ocellated Turkey (*Meleagris ocellata*). Ithaca, NY, USA: Cornell Lab of Ornithology; 2012 [cited 2015 August 8, 2015]. Available from: <http://neotropical.birds.cornell.edu/portal/species/overview?p_p_spp=83431>.

15. Rasmussen PC. AV#18329: Ocellated turkey: *Meleagris ocellata*. East Lansing, MI, USA: Avian Vocalization Center, Michigan State University; 2014 [cited 2015 August 30]. Available from: <http://avocet.zoology.msu.edu/recordings/18329>.

16. Macauley LR. ML102559: Ocellated turkey: *Meleagris ocellata*. Ithaca, NY, USA: Macauley Library, Cornell Lab of Ornithology; [cited 2015 August 30]. Available from: https://macaulaylibrary.org/audio/102559.
