# Supplementary material for: Biomechanics of the Peacock’s Display: How Feather Structure and Resonance Influence Multimodal Signaling
Source: PLoS One. 2016 Apr 27;11(4):e0152759. doi: 10.1371/journal.pone.0152759 (PMC4847759; doi:10.1371/journal.pone.0152759)
Supplement: S3 Text — (DOCX) [file pone.0152759.s008.docx]

**S3 Text. Audio analysis and characterization of sound production**

We computed the Fourier spectrum for 1.00 s duration audio waveforms from train-rattling sequences for three intense bouts performed by each of 10 males. We then identified the frequency of 2-5 peaks with the highest FFT magnitude above noise and below 55 Hz (because the audio recordings had prominent background noise above 55 Hz). We used a histogram of all *n* = 116 peaks detected in this way to identify the two most prominent peaks at approximately 26 and 52 Hz (Fig S2A). We then computed the means for each of these two peaks for each male as well as the grand means for all 10 males (grand means = 25.6 Hz [24.7, 26.5] and 51.0 Hz [50.3, 51.7]). We also examined Fourier spectra from six background recordings made between sequences of train-rattling to confirm that no peaks at ~26 Hz or 52 Hz were apparent in the absence of displaying.

To determine the locus of sound production, we manually scanned a microphone approximately 20 cm behind the train and tail feathers while a peacock was performing the train-rattling display. At the same time, we made a video recording of the microphone and the displaying male to identify the microphone location and to record the sound from a fixed location. Nine recordings were measured and analyzed (3 each for 3 males; Fig S2B-C). In every case, the sound waveform amplitude in the audio recording was at a maximum when the microphone was immediately behind the tail and decreased as the microphone was moved toward the distal end of the train feathers.

**
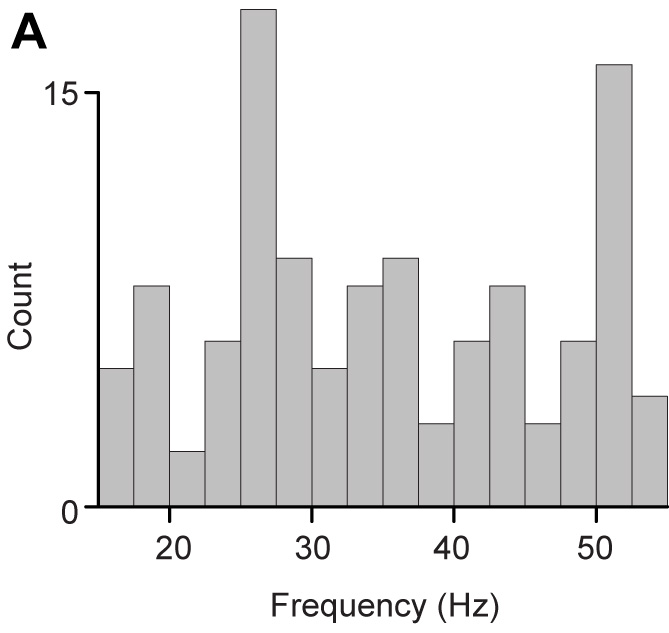
**


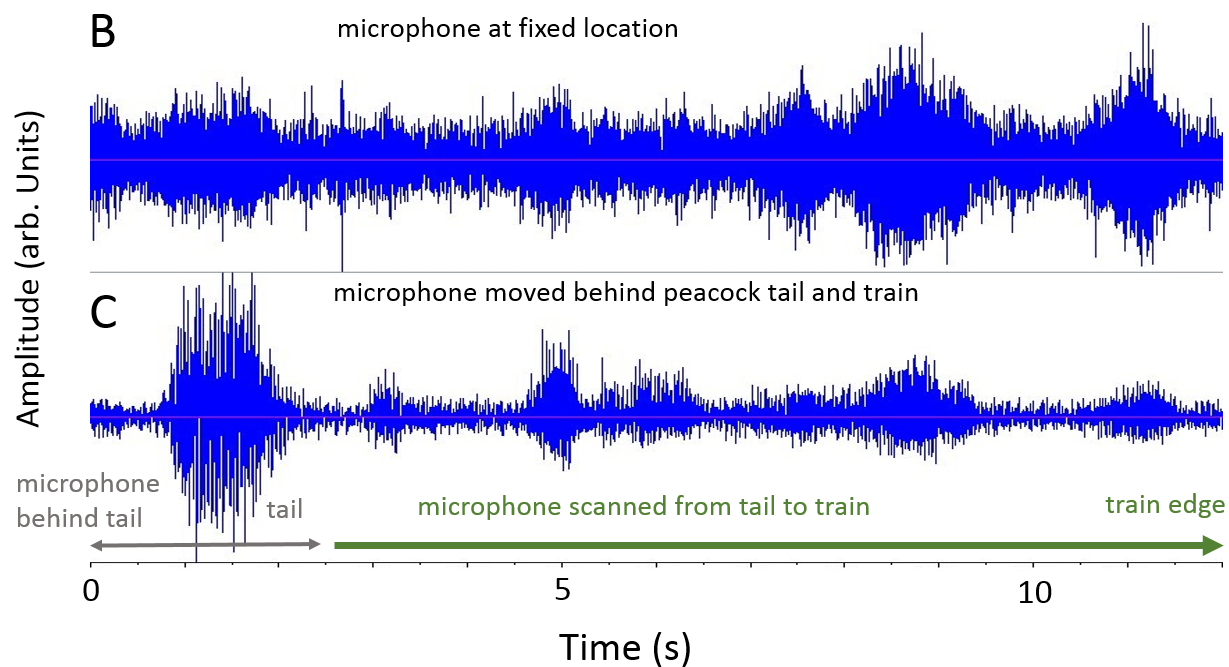


**Fig S2. Peak frequencies of peacock train-rattling and waveforms illustrating the enhanced sound intensity emitted near the tail.** (A) Histogram of peak frequencies in field audio recordings of peacock train-rattling displays (*n* =116 spectral peaks from 30 recordings of 10 adult peacocks). The most common peak frequencies are at approximately 26 and 52 Hz, in agreement with the average train-rattling frequency and two times that frequency. (B) and (C) show waveforms recorded in the field while a peacock performed repeated pulses of train-rattling. The waveform in (B) was recorded by the camcorder used to record the video, which was at a fixed location. The waveform in (C) was recorded using a microphone held in close proximity to the rear of the displaying male and scanned from a position behind the tail to a position behind the distal edge of the train. Relatively constant amplitudes were recorded by the fixed microphone, whereas the moving microphone recorded higher amplitudes behind the tail than behind the train feathers, demonstrating that sound emission per unit area was most intense immediately behind the tail. Note that the higher background noise level in (B) is due to the lower quality of the camcorder audio recording relative to the professional quality audio equipment described in the main text, which was used to record (C).
