# Supplementary material for: Biomechanics of the Peacock’s Display: How Feather Structure and Resonance Influence Multimodal Signaling
Source: PLoS One. 2016 Apr 27;11(4):e0152759. doi: 10.1371/journal.pone.0152759 (PMC4847759; doi:10.1371/journal.pone.0152759)
Supplement: S4 Text — (DOCX) [file pone.0152759.s009.docx]

**S4 Text. Calculation of mechanical properties of the feather rachis**

To compute the second moment of area, *I*, for the feather rachis cross-section, we modeled each short segment of rachis as an approximately cylindrical keratin cortex shell of thickness *d* in the lateral direction filled with a keratin pith core with densities *ρ_s_* and *ρ_c_*, respectively. The cross-section of the rachis was modeled as an ellipse [1] with axis dimensions *D_L_* and *D_D_*, where the subscripts refer to the lateral (side-to-side) and dorsoventral (front-to-back) planes respectively. The thickness of the dorsoventral shell walls was assumed to be twice that of the lateral walls based on the rachis cross-sectional geometries in previous studies of feather biomechanical properties [1-3]. We used these values to find *d* by solving the resulting quadratic equation for rachis linear density:

$\mu_{R}=\frac{\pi}{4}\left(\rho_{s}D_{L}D_{D}+\left(\rho_{c}-\rho_{s}\right)\left(D_{L}-2d\right)\left(D_{D}-4d\right)\right)$ . (1)

This allowed us to compute the lateral shell thickness, *d*, for each relative rachis position, *u* = *x/L_R_*, from our empirically determined values of *μ_R_(u)*, *D_L_(u)* and *D_D_(u).* Because the cortex shell dominates the bending stiffness of the rachis [4, 5], the second moments of area for bending in the lateral and dorsoventral planes, *I_L_(u)* and *I_D_(u)*, respectively, were computed as that of a cortex shell with an elliptical cross-section using [6]:

$I_{L}=\frac{\pi}{64}\left(D_{D}D_{L}^{3}-\left(D_{D}-4d\right)\left(D_{L}-2d\right)^{3}\right)$ (2a)

$I_{D}=\frac{\pi}{64}\left(D_{L}D_{D}^{3}-\left(D_{L}-2d\right)\left(D_{D}-4d\right)^{3}\right)$ . (2b)

Schmitz et al. showed that profiles of the rachis second moment of area calculated from cross-sectional geometry agree closely with those measured using bending experiments [2]. Calculations of feather resonant frequency used *I* = *I_L_*, since train-rattling involves lateral oscillations.

We used our measured rachis diameters and lengths along with calculated second moment of area values to compute the ratio between the critical stresses for local and Euler buckling, *σ_L_* and *σ_E_*, respectively:

$1\leq\frac{\sigma_{L}}{\sigma_{E}}=\frac{k A_{s} d L_{R}^{2}}{n_{b} \pi^{2}D_{j}I_{j}}\approx\frac{0.27A_{s} d L_{R}^{2}}{D_{j}I_{j}}$ (3)

where *n_b_* = 0.25, *k* ≈ 0.67 [1, 7] and *j* = *L* (lateral) or *D* (dorsoventral). The inequality in this equation tests whether the feather will bend elastically rather than irreversibly buckling locally (i.e., developing a kink).

We obtained the cortex shell and core densities from linear fits to Fig 3 in [4]. The density values from [1] gave the same second moment of area profiles with an overall change in magnitude of 18%, but poorer agreement with published values for shell thickness. The standard errors in *I* and other computed quantities were estimated from measurements taken from four eyespot feathers of each length, as described in the main text.

Our calculated *d*(*u*) values agreed with those measured in [4] and with those calculated from data reported in [1]. Our computed core area:shell area ratio for *L* = 1.00 m was 9.3 ± 3.4 (mean ± s.d.), in agreement with the value of 9.0 reported in [1]. Similar ratios of *σ*_L_ / *σ*_E_ result from using dimensions measured microscopically for *L* = 1 m eyespot feather [4]. However, it should be noted that our model assumes a consistently elliptical cross-section, and ignores the more complex internal structure noted in [1] (e.g., the presence of a thickened rib running along the ventral side of the rachis near the proximal end, as well as internal stiffening septa).

**References**

1. Liu ZQ, Jiao D, Meyers MA, Zhang ZF. Structure and mechanical properties of naturally occurring lightweight foam-filled cylinder – The peacock’s tail coverts shaft and its components. Acta Biomaterialia. 2015;17:137-51.

2. Schmitz A, Ponitz B, Bruecker C, Schmitz H, Herweg J, Bleckmann H. Morphological properties of the last primaries, the tail feathers, and the alulae of *Accipiter nisus*, *Columba livia*, *Falco peregrinus*, and *Falco tinnunculus*. Journal of Morphology. 2015;276(1):33-46. doi: 10.1002/jmor.20317. PubMed PMID: WOS:000347054600003.

3. Purslow PP, Vincent JFV. Mechanical properties of primary feathers from pigeons. Journal of Experimental Biology. 1978;72(FEB):251-60. PubMed PMID: WOS:A1978EM51700017.

4. Weiss IM, Kirchner HOK. The peacock's train (*Pavo cristatus* and *Pavo cristatus mut. alba*) I. structure, mechanics, and chemistry of the tail feather coverts. Journal of Experimental Zoology Part a-Ecological Genetics and Physiology. 2010;313A(10):690-703. doi: 10.1002/jez.641. PubMed PMID: WOS:000284215000006.

5. Purslow PP, Vincent JFV. Mechanical properties of primary feathers from the pigeon. Journal of Experimental Biology. 1978;72(FEB):251-60. PubMed PMID: WOS:A1978EM51700017.

6. Beer FP, Johnston J, Russel. Vector mechanics for engineers. New York, NY: McGraw-Hill; 1984.

7. Wainwright SA, Biggs WD, Currey JD, Gosline JM. Mechanical design in organisms. Princeton, NJ USA: Princeton University Press; 1982.
