# Supplementary material for: Biomechanics of the Peacock’s Display: How Feather Structure and Resonance Influence Multimodal Signaling
Source: PLoS One. 2016 Apr 27;11(4):e0152759. doi: 10.1371/journal.pone.0152759 (PMC4847759; doi:10.1371/journal.pone.0152759)
Supplement: S5 Text — (DOCX) [file pone.0152759.s010.docx]

**S5 Text. Feather vibrational resonance**

Additional laboratory shaking experiments were performed on one *L_R_* ≈ 112 cm eyespot feather and three different length rectrices mounted in a viscoelastic polymer (Composimold Corp., Manchester, ME, USA) to simulate connective tissue and muscle [1]. Mounting these feathers in a viscoelastic gel reduced resonant frequencies only slightly and significantly damped out oscillations above 36 - 40 Hz compared with experiments using rigid mounts (Fig S3). The lack of a substantial difference at frequencies < 30 Hz supports our use of rigid mounts to evaluate resonance at display frequencies.


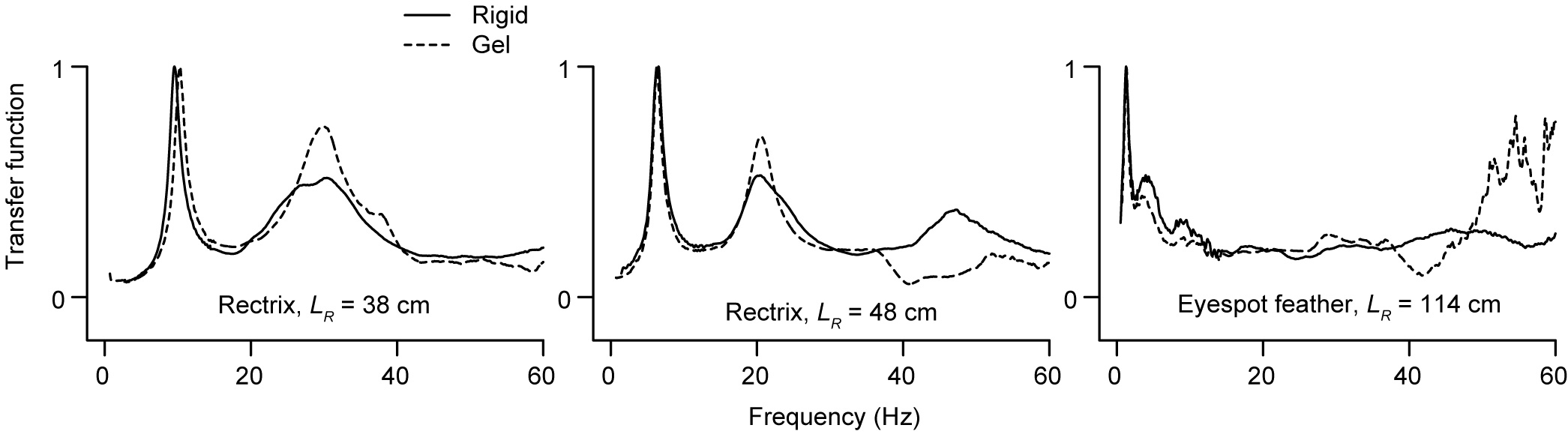


**Fig S3. Examples of vibrational responses derived when feathers were shaken using two different mounting methods (rigid holder vs viscoelastic gel).** Each panel shows results for a single feather tested in both conditions.

The amplitude *A* of vibrations driven at frequency *f_d_* for a mechanical resonator with one degree of freedom is predicted to follow a Lorentzian spectral response:

$H\left(f_{d}\right)=\frac{A}{A_{d}}=\frac{f_{n}/f_{d}}{\sqrt{\left(f_{n}/f_{d}-f_{d}/f_{n}\right)^{2}+\frac{1}{Q_{n}^{2}}}}$ (1)

where *f_k_* is the natural frequency and *Q_k_* is the quality factor [2]. This form of the transfer function, *H(f_d_)*, was used to fit our spectral response data using nonlinear least squares fitting, since it allows direct computation of the standard errors of both *f_k_* and *Q_k_*. Because we used a frequency sweep to stimulate the feathers during feather shaking experiments, we analyzed the resulting data (tip or eyespot position vs time) using the spectrogram function in Matlab.

Power laws were fitted to the rectrix resonant frequency vs *L_R_* data without log transformation because there was no reason to expect errors to add multiplicatively [3]. Fits to the rectrix data used all available resonant peak data for both rigid- and gel-mounted feathers because the mounting method had minimal effect on the frequencies of interest.

Fig S4 shows plots of the length dependence of the quality factor, *Q*, measured on single feathers and discussed in the main text.


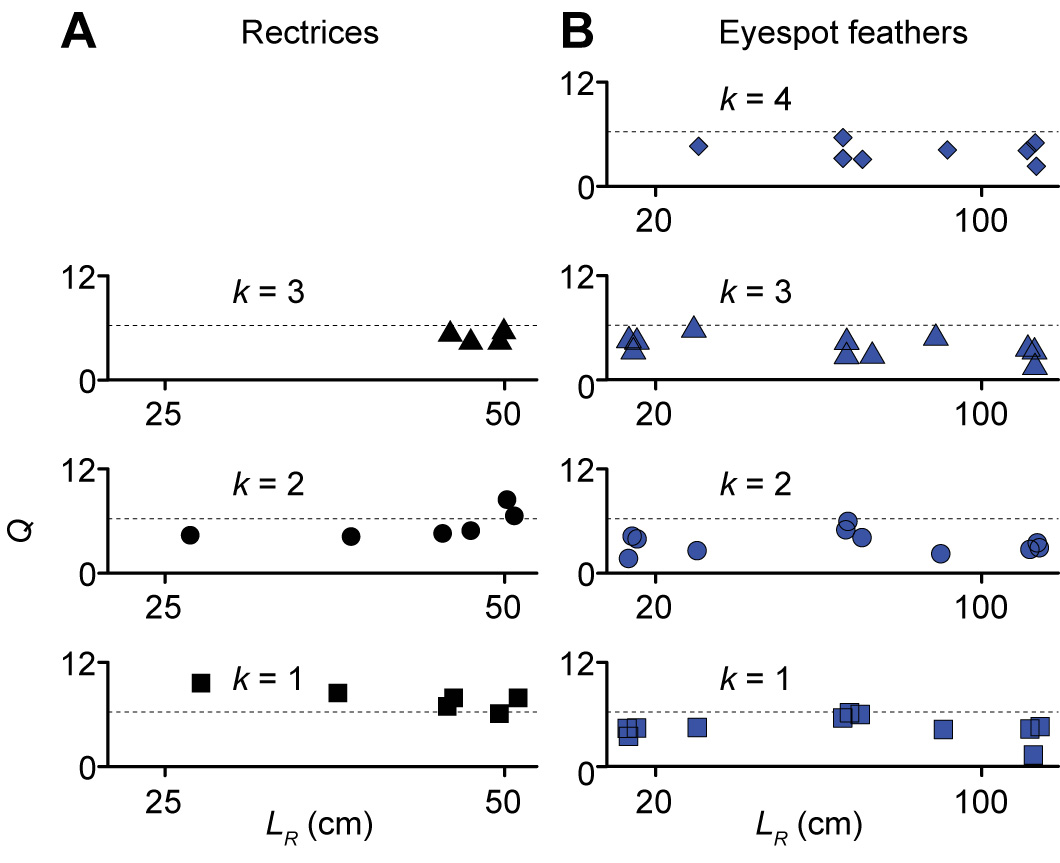


**Fig S4. Quality factor, *Q_k_*, measured for each *k*th vibrational mode from single feathers.** Results for (A) rectrices and (B) eyespot feathers. Lower *Q_k_* values indicate more damping, and horizontal dotted lines at *Q_k_* = 2π (for which stored elastic energy = energy dissipated per cycle) approximately divide damping regimes. Note that the x-axis is scaled differently in (A) and (B).

**Table A.** **Feathers used in laboratory shaking experiments.**

| feather type | length *L* (cm) | rachis length *L_R_* (cm) | rigid mount | gel mount |
| --- | --- | --- | --- | --- |
| single eyespot feathers | 19.3 * | 14.2 | X |  |
|  | 19.3 | 13.5 | X |  |
|  | 19.3 | 13.6 | X |  |
|  | 36.5 * | 30.0 | X |  |
|  | 79.6 * | 66.6 | X |  |
|  | 79.8 | 70.5 | X |  |
|  | 79.3 | 67.1 | X |  |
|  | 78.5 | 68 | X |  |
|  | 103.5 * | 90.2 | X |  |
|  | 126.5 * | 112.0 | X |  |
|  | 126.3 | 114.0 | X | X |
|  | 126.9 | 112.6 | X |  |
| eyespot manipulation (unaltered, mass added, eyespot removed) | 127.3 * | 111.0 | X |  |
| single male rectrix (tail) feathers | 30.6 * | 27.0 | X |  |
|  | 42.1 * | 38.2 | X | X |
|  | 41.7 | 37.6 |  | X |
|  | 47.4 * | 43.0 |  | X |
|  | 48.9 * | 45.2 | X |  |
|  | 51.2 * | 48.0 | X | X |
|  | 52.9 | 50.6 | X |  |
|  | 52.0 * | 50.0 | X |  |
| single female tail covert feathers | 31.7 * | 29.2 | X |  |
|  | 31.5 | 29.2 |  |  |
|  | 31.3 | 29.2 | X |  |
| tail feather array * | 46.0, 48.2, 49.1, 50.2, 52.1 | 46.0, 48.2, 49.1, 50.2, 52.1 | X |  |
| female tail covert array * | 23.5, 22.0, 23.5, 20.7, 24.0 | 23.5, 22.0, 23.5, 20.7, 24.0 | X |  |
| train feather array * (feather in columns listed front to rear) Note: 80% of these feathers were obtained from a single healthy adult male killed by another animal in a zoo. | left column eyespots: 22.4, 34.3, 50.1, 63.5, 88.1, 102.6, 125.3, 131.0 | 15.2, 27.6, 42.4, 53.5, 76.5, 89.8, 112.5, 118.5 |  | X |
|  | central column eyespots: 27.7, 48.0, 78.0, 95.3, 112.3, 125.4, 138.5 | 20.7, 42.2, 65.0, 82.4, 109.0, 110.8, 127.2 |  |  |
|  | right column eyespots: 22.4, 37.0, 68.2, 90.0, 102.8, 115.6, 121.0, 137.4 | 14.6, 28.9, 58.2, 76.6, 90.4, 101.5, 110.6, 125.3 |  |  |
|  | fishtail feathers (at rear): 122.5. 133.0 | 116, 126.6 |  |  |

Shaking spectra and related measurements were reproduced three times for samples marked with an asterisk (*).

**References**

1. Park J, Lakes RS. Biomaterials: an introduction. New York, NY, USA: Springer; 2007.

2. Smith WF. Waves and oscillations: a prelude to quantum mechanics. Oxford, UK: Oxford University Press; 2010.

3. Niklas KJ, Hammond ST. Assessing scaling relationships: uses, abuses and alternatives. International Journal of Plant Sciences. 2014;175(7):754-63. doi: 10.1086/677238. PubMed PMID: WOS:000341514700003.
