# Supplementary material for: Biomechanics of the Peacock’s Display: How Feather Structure and Resonance Influence Multimodal Signaling
Source: PLoS One. 2016 Apr 27;11(4):e0152759. doi: 10.1371/journal.pone.0152759 (PMC4847759; doi:10.1371/journal.pone.0152759)
Supplement: S6 Text — (DOCX) [file pone.0152759.s011.docx]

**S6 Text. Comparison of eyespot motion and peafowl visual acuity**

To determine the angular extent of eyespot motion on the peahen’s visual field, we reviewed our field videos to find close-up views with the greatest vibrational amplitude during train rattling, and used these to track one eyespot at the train’s outer edge and one eyespot in the middle of the train near the tips of the centermost tail feathers. The tracked values of eyespot pixel coordinates vs time were then Fourier analyzed, revealing a clear peak at approximately 26 Hz. These data were then smoothed over 19 frames to remove slow variations due to the male tilting his train toward the female and/or moving to face her. We verified that this smoothing procedure did not affect the magnitude of the main peak at approximately 26 Hz, and checked that there were not significant Fourier components above 90 Hz, the critical flicker frequency for domestic chickens *Gallus domesticus* [1]. Although the critical flicker fusion has not been measured for peafowl, domestic chickens are in the same order as peafowl (*Galliformes*) and have similar foraging behaviour, suggesting that their visual systems may be similar.

The smoothed coordinates of moving eyespots were analyzed to find the median peak-to-peak eyespot displacement in pixels, *w_E_*, which was then converted into millimeters using previously measured diameters of the bronze part of the eyespots (40.5 mm [38.5, 42.5 mm]; data from Dakin and Montgomerie 2013). For comparison, we also obtained peak-to-peak amplitudes of oscillatory motion of the tips of loose barbs near the same eyespots that were tracked in the middle of the train. In a few cases the video was not of sufficient quality for the loose barbs to be resolved.

As a benchmark for interpreting these results, we used ν_s =_ 20.6 cycles deg^-1^ as an upper bound on peafowl spatial acuity, based on retinal cell densities and visual anatomy of 1-year-old peacocks [2]. This measure of acuity can be converted to the angular extent, *Δφ_s_*, of the smallest visually resolvable feature using *Δφ_s_* = 1/ν_s_ = (π/180 deg)/(20.6 cycles deg^-1^) = 8.47 ×10^-4^ radians. This angular extent is related to the size of the smallest visually resolvable feature (*w*) at a distance (*z*) by: *Δφ_s_* = *w/z* [3]. Visual acuities derived from this theoretical retinal anatomy are typically slight overestimates of actual visual acuities derived from behavioural tests [2, 4, 5].

**References**

1. Lisney TJ, Rubene D, Rozsa J, Lovlie H, Hastad O, Odeen A. Behavioural assessment of flicker fusion frequency in chicken *Gallus gallus domesticus*. Vision Research. 2011;51(12):1324-32. doi: 10.1016/j.visres.2011.04.009. PubMed PMID: WOS:000291778400004.

2. Hart NS. Vision in the peafowl (Aves: *Pavo cristatus*). Journal of Experimental Biology. 2002;205(24):3925-35. PubMed PMID: WOS:000180206200016.

3. Land MF, Nilsson DE. Animal eyes: Oxford University Press; 2012. 271 p.

4. Ghim MM, Hodos W. Spatial contrast sensitivity of birds. Journal of Comparative Physiology a-Neuroethology Sensory Neural and Behavioral Physiology. 2006;192(5):523-34. doi: 10.1067/s00359-005-0090-5. PubMed PMID: WOS:000237947300010.

5. Reymond L. Spatial visual acuity of the eagle *Aquila audax*: a behavioral, optical and anatomical investigation. Vision research. 1985;25(10):1477-91. doi: 10.1016/0042-6989(85)90226-3.
