# Supplementary material for: Biomechanics of the Peacock’s Display: How Feather Structure and Resonance Influence Multimodal Signaling
Source: PLoS One. 2016 Apr 27;11(4):e0152759. doi: 10.1371/journal.pone.0152759 (PMC4847759; doi:10.1371/journal.pone.0152759)
Supplement: S7 Text — (DOCX) [file pone.0152759.s012.docx]

**S7 Text. Statistical models of morphology and train-rattling frequency**

To examine the factors predicting the vibration frequency of feathers during the train-rattling displays by adult peacocks, we evaluated 13 candidate linear mixed-effects models (LMEMs) using the *dredge* function in the *MuMIn* package in R. We constructed this candidate model set from the following potential predictors: temporal variables (sample interval, time of day, and day of year), morphological traits (tail length, train length, and the number of eyespots), and air temperature. Because we had only 12 individuals and their morphological traits were correlated, we considered at most one morphological trait per candidate model to minimize the effects of collinearity and overparameterization. Additionally, because temperature was associated with time of day, we considered at most one of either temperature or time of day per candidate model. We also included the intercept-only model in our candidate model set (i.e., the model with no fixed effect predictors). All candidate models are listed in Table A. We accounted for nonindependence of repeated samples by nesting display bouts within individuals as a random effect.

This analysis revealed two top models, with ∆AICc < 2. Both models included train length as a predictor of feather vibration frequency, but the second-ranked model did not include time of day as a predictor (Table A). We averaged only the two top models using the *model.avg* function in *MuMIn*, and we report this averaged model in the main text.

Repeating this analysis using a model that did not correct for train growth gave the same results, except that there are 3 top models with ∆AICc < 2 and the third ranked model includes the number of eyespots as an additional predictor (Table B).

**Table A.** **Fitted models of adult peacock train-rattling vibration frequency in relation to temporal variables, morphological traits, and air temperature (*n* = 103 observations of 35 displays by 12 individuals).**

|  | estimates | | | | | | | |  |  |  |  |
| --- | --- | --- | --- | --- | --- | --- | --- | --- | --- | --- | --- | --- |
|  |  | temporal predictors | | |  | morphological predictors | | |  |  |  |  |
| model rank # | intercept (Hz) | sample interval | time of day (hour) | day of year (day) | temperature (ºC) | # eyespots | tail length (cm) | train length* (cm) | df | AICc | ∆AICc | Akaike weight |
| 1 | 26.98 | + | 0.08 | -0.10 | -- | -- | -- | 0.04 | 9 | 286.81 | 0 | 0.39 |
| 2 | 28.82 | + | -- | -0.11 | -- | -- | -- | 0.04 | 8 | 288.11 | 1.31 | 0.20 |
| 3 | 20.26 | + | 0.10 | -0.09 | -- | 0.07 | -- | -- | 9 | 288.92 | 2.12 | 0.13 |
| 4 | 22.47 | + | 0.11 | -0.07 | -- | -- | 0.15 | -- | 9 | 290.22 | 3.41 | 0.07 |
| 5 | 27.36 | + | -- | -0.10 | 0.02 | -- | -- | 0.04 | 9 | 290.36 | 3.55 | 0.07 |
| 6 | 30.59 | + | 0.10 | -0.08 | -- | -- | -- | -- | 8 | 291.26 | 4.45 | 0.04 |
| 7 | 23.16 | + | -- | -0.10 | -- | 0.07 | -- | -- | 8 | 291.45 | 4.64 | 0.04 |
| 8 | 33.31 | + | -- | -0.10 | -- | -- | -- | -- | 7 | 293.08 | 6.28 | 0.02 |
| 9 | 26.41 | + | -- | -0.09 | -- | -- | 0.12 | -- | 8 | 293.15 | 6.35 | 0.02 |
| 10 | 20.39 | + | -- | -0.08 | 0.04 | 0.07 | -- | -- | 9 | 293.34 | 6.53 | 0.01 |
| 11 | 21.79 | + | -- | -0.05 | 0.06 | -- | 0.13 | -- | 9 | 294.61 | 7.80 | 0.01 |
| 12 | 30.15 | + | -- | -0.07 | 0.04 | -- | -- | -- | 8 | 294.90 | 8.09 | 0.01 |
| 13 | 25.36 | -- | -- | -- | -- | -- | -- | -- | 4 | 310.22 | 23.41 | 0 |

* corrected for growth

Sample interval was a categorical predictor and had three levels: peak amplitude, pre-peak, and post-peak, with reference to when the vibration frequency was sampled during the display. The estimates provided for continuous predictors are the rate of change of vibration frequency for one unit change in the predictor when the other predictor variables are fixed. The corrected Akaike’s Information Criterion (AICc) is a measure of relative goodness-of-fit of different models. ∆AICc is the difference in AICc between each model and the best-fitting model. Models with ∆AICc ≤ 2 are considered to have equivalent support. Akaike weight is the likelihood that a model is best fit relative to the others in the set.

**Table B.** **Fitted models of adult peacock train-rattling vibration frequency without correcting for train growth (all other details as in Table A).**

|  | estimates | | | | | | | |  |  |  |  |
| --- | --- | --- | --- | --- | --- | --- | --- | --- | --- | --- | --- | --- |
|  |  | temporal predictors | | |  | morphological predictors | | |  |  |  |  |
| model rank # | intercept (Hz) | sample interval | time of day (hour) | day of year (day) | temperature (ºC) | # eyespots | tail length (cm) | train length** (cm) | df | AICc | ∆AICc | Akaike weight |
| 1 | 25.75 | + | 0.08 | -0.08 | -- | -- | -- | 0.04 | 9 | 286.95 | 0 | 0.36 |
| 2 | 27.41 | + | -- | -0.10 | -- | -- | -- | 0.04 | 8 | 287.96 | 1.01 | 0.22 |
| 3 | 20.26 | + | 0.10 | -0.09 | -- | 0.07 | -- | -- | 9 | 288.92 | 1.97 | 0.14 |
| 4 | 22.47 | + | 0.11 | -0.07 | -- | -- | 0.15 | -- | 9 | 290.22 | 3.26 | 0.07 |
| 5 | 26.33 | + | -- | -0.09 | 0.02 | -- | -- | 0.04 | 9 | 290.28 | 3.32 | 0.07 |
| 6 | 30.59 | + | 0.10 | -0.08 | -- | -- | -- | -- | 8 | 291.26 | 4.30 | 0.04 |
| 7 | 23.16 | + | -- | -0.10 | -- | 0.07 | -- | -- | 8 | 291.45 | 4.49 | 0.04 |
| 8 | 33.31 | + | -- | -0.10 | -- | -- | -- | -- | 7 | 293.08 | 6.13 | 0.02 |
| 9 | 26.41 | + | -- | -0.09 | -- | -- | 0.12 | -- | 8 | 293.15 | 6.2 | 0.02 |
| 10 | 20.39 | + | -- | -0.08 | 0.04 | 0.07 | -- | -- | 9 | 293.34 | 6.38 | 0.01 |
| 11 | 21.79 | + | -- | -0.05 | 0.06 | -- | 0.13 | -- | 9 | 294.61 | 7.66 | 0.01 |
| 12 | 30.15 | + | -- | -0.07 | 0.04 | -- | -- | -- | 8 | 294.90 | 7.95 | 0.01 |
| 13 | 25.36 | -- | -- | -- | -- | -- | -- | -- | 4 | 310.22 | 23.27 | 0 |

** raw measurement, not adjusted for growth between the date the train was measured and the date the display was video-recorded
