# Supplementary material for: Biomechanics of the Peacock’s Display: How Feather Structure and Resonance Influence Multimodal Signaling
Source: PLoS One. 2016 Apr 27;11(4):e0152759. doi: 10.1371/journal.pone.0152759 (PMC4847759; doi:10.1371/journal.pone.0152759)
Supplement: S8 Text — (DOCX) [file pone.0152759.s013.docx]

**S8 Text. Model of resonant properties of the tail**

We approximated the resonant response of the entire tail (excluding the train) by modeling it as an array of independently vibrating rectrices with the properties derived from our laboratory feather-shaking experiments. This model assumes that there are only weak interactions among the tail feathers, between the tail and train feathers, and between the tail and the peacock’s body. To estimate the drive power transferred to the tail during shaking at each drive frequency, *f_d_*, at a given driving force, *F_o_*, we summed the drive power required to shake each feather in isolation [1]:

 (1)

where the sum over *i* runs over all *N_F_* feathers in the array of rectrices, the sum over *k* runs over all normal modes, and *μ_Fi_* is average feather linear density of the *i*^th^ feather [2]. For the 12 males from which we obtained tail morphology data, we measured the lengths of all rectrices in the tail *in vivo*. We then measured the average linear density of 17 male rectrices representing 95% of the rectrix lengths found in tails measured in the field, and used these measurements to compute rectrix *μ_Ri_* as a function of length. Results from our shaking measurements on single tail feathers were used to calculate values for *f_ik_* and *Q_ik_*.

Error bars in the peak resonant frequencies and *Δf_3dB_* for the model tail were computed from fits to the computed power spectrum and from the spread in the measured values of average rectrix linear density.

**References**

1. Smith WF. Waves and oscillations:  a prelude to quantum mechanics. Oxford, UK: Oxford University Press; 2010.

2. Neimark MA, Andermann ML, Hopfield JJ, Moore CI. Vibrissa resonance as a transduction mechanism for tactile encoding. Journal of Neuroscience. 2003;23(16):6499-509. PubMed PMID: WOS:000184323900013.
