# Supplementary material for: Biomechanics of the Peacock’s Display: How Feather Structure and Resonance Influence Multimodal Signaling
Source: PLoS One. 2016 Apr 27;11(4):e0152759. doi: 10.1371/journal.pone.0152759 (PMC4847759; doi:10.1371/journal.pone.0152759)
Supplement: S9 Text — (PDF) [file pone.0152759.s014.pdf]

Statistical Supplement

Table of Contents

Setup 1
R details 1
Packages to load 1
Datasets 2
Descriptive statistics for peacock displays 2
Comparing train- and covert-rattling frequencies 3
Peacock train growth analysis 10
Factors associated with peacock vibration frequency 14

This is the R code and output for statistical analyses included in this paper and supplementary material: *Dakin R, McCrossan O, Hare JF, Montgomerie R, Kane SA. 2015 ms Biomechanics of the peacock's display: how feather structure facilitates the performance of a multimodal signal*

Setup

R details

- File creation date: 2015-12-20
- R version 3.2.3 (2015-12-10)
- `dplyr` package version: 0.4.3
- `nlme` package version: 3.1.122
- `lme4` package version: 1.1.10
- `MuMIn` package version: 1.15.1
- `multcomp` package version: 1.4.1
- `boot` package version: 1.3.17
- `multcomp` package version: 1.4.1

Packages to load

```
library(dplyr)
library(nlme)
library(lme4)
library(MuMIn)
library(multcomp)
library(boot)
```

```
library(car)
library(psych)
library(Rmisc)
library(arm)
```

Datasets

The data analyzed here are in the following datasets, which will be deposited on DRYAD:

- growth.csv: train feather growth during the breeding season
- rattle.csv: train and train-rattling data
- other.csv: wing-shaking and shivvering data

Load the data into R from the active workspace.

```
growth <-  read.csv("growth.csv")
rattle <-  read.csv("rattle.csv")
other <-  read.csv("other.csv")
```

Descriptive statistics for peacock displays

Summarized in table 1 of the main text.

```
peak <- subset(rattle, sample=='peak') #select data for peak period

#analysis of peak frequencies for train- (male) and covert-rattling frequenci
es (female)
idmeans0 <- aggregate(peak$freq, by=list(id=peak$id,type=peak$type), 'mean')
#calculate mean peak frequency for each individual
describeBy(idmeans0$x, group=idmeans0$type) #decriptive stats for peak freque
ncy each age/sex category calculated from means per individual

## group: female
##   vars n  mean   sd median trimmed  mad   min  max range skew kurtosis
## 1    1 5 26.11 0.69  26.08   26.11 0.42 25.24 27.1  1.86 0.17    -1.62
##     se
## 1 0.31
## --------------------------------------------------------
## group: juvenile
##   vars  n  mean   sd median trimmed  mad   min   max range skew kurtosis
## 1    1 14 26.47 1.35  26.11   26.37 1.35 24.88 29.24  4.36 0.56    -0.98
##     se
## 1 0.36
## --------------------------------------------------------
## group: male
##   vars  n  mean   sd median trimmed  mad   min   max range  skew kurtosis
## 1    1 14 25.59 0.94  25.53   25.61 1.13 23.91 27.05  3.13 -0.04    -1.36
##     se
## 1 0.25
```

```
#confidence intervals for each of the means in descriptive stats above
CI(subset(idmeans0$x, idmeans0$type=='female'))

##    upper     mean    lower 
## 26.96664 26.11200 25.25736

CI(subset(idmeans0$x, idmeans0$type=='male'))

##    upper     mean    lower 
## 26.13215 25.58821 25.04428

CI(subset(idmeans0$x, idmeans0$type=='juvenile'))

##    upper     mean    lower 
## 27.25093 26.47054 25.69014

#analysis of shivers and wing shakes from 'others' dataset
idmeans1 <- aggregate(other$frequency, by=list(id=other$id,behaviour=other$be
haviour), 'mean') #this calculates mean frequency of each behaviour for each 
individual

describeBy(idmeans1$x, group = idmeans1$behaviour) #calculate separate descri
ptive stats for shiver and wing-shake

## group: shiver
##   vars n  mean   sd median trimmed  mad  min   max range  skew kurtosis
## 1    1 7 10.39 0.34  10.59   10.39 0.15 9.79 10.69   0.9 -0.65    -1.41
##     se
## 1 0.13
## -------------------------------------------------------- 
## group: wing-shake
##   vars  n mean   sd median trimmed  mad  min  max range skew kurtosis   se
## 1    1 11 5.43 0.77   5.16    5.39 0.86 4.46 6.75   2.3 0.36    -1.44 0.23

#confidence intervals for each of the means in descriptive stats above
CI(subset(idmeans1$x, idmeans1$behaviour =='shiver'))

##    upper     mean    lower 
## 10.70867 10.39024 10.07181

CI(subset(idmeans1$x, idmeans1$behaviour =='wing-shake'))

##    upper     mean    lower 
## 5.946170 5.425866 4.905561
```

Comparing train- and covert-rattling frequencies

Compare age/sex classes during pre-peak, peak and post-peak samples

```
#full model with interaction term
mod3 <- lme(freq~sample*type, random=~1|id/boutID, data=rattle, na.action=na.
```

```
omit)
summary(mod3)
```

```
## Linear mixed-effects model fit by REML
##  Data: rattle
##        AIC      BIC    logLik
##   687.8822 728.7143 -331.9411
##
## Random effects:
##  Formula: ~1 | id
##         (Intercept)
## StdDev:   0.9632442
##
##  Formula: ~1 | boutID %in% id
##         (Intercept) Residual
## StdDev:   0.7756886 0.723754
##
## Fixed effects: freq ~ sample * type
##                              Value Std.Error  DF  t-value p-value
## (Intercept)              26.151031 0.5786406 145 45.19391  0.0000
## samplepost                0.380551 0.4118299 145  0.92405  0.3570
## samplepre                 0.983267 0.4402705 145  2.23332  0.0271
## typejuvenile              0.203655 0.6652210  30  0.30615  0.7616
## typemale                 -0.562115 0.6553780  30 -0.85770  0.3979
## samplepost:typejuvenile -0.743627 0.4498251 145 -1.65315  0.1005
## samplepre:typejuvenile  -0.591074 0.4784853 145 -1.23530  0.2187
## samplepost:typemale     -1.010485 0.4424872 145 -2.28365  0.0238
## samplepre:typemale      -0.984997 0.4695673 145 -2.09767  0.0377
##  Correlation:
##                         (Intr) smplps smplpr typjvn typeml smplpst:typj
## samplepost              -0.247
## samplepre               -0.231  0.454
## typejuvenile            -0.870  0.215  0.201
## typemale                -0.883  0.218  0.204  0.768
## samplepost:typejuvenile  0.226 -0.916 -0.416 -0.251 -0.200
## samplepre:typejuvenile   0.212 -0.418 -0.920 -0.236 -0.188  0.458
## samplepost:typemale      0.230 -0.931 -0.422 -0.200 -0.248  0.852
## samplepre:typemale       0.217 -0.426 -0.938 -0.188 -0.234  0.390
##                         smplpr:typj smplpst:typm
## samplepost
## samplepre
## typejuvenile
## typemale
## samplepost:typejuvenile
## samplepre:typejuvenile
## samplepost:typemale      0.389
## samplepre:typemale       0.863       0.459
##
## Standardized Within-Group Residuals:
##         Min          Q1         Med          Q3         Max
```

```
## -2.80198739 -0.46673180 -0.06967542  0.41924303  2.30291166
##
## Number of Observations: 231
## Number of Groups:
##             id boutID %in% id
##             33              80
```

```
Anova(mod3)
```

```
## Analysis of Deviance Table (Type II tests)
##
## Response: freq
##                Chisq Df Pr(>Chisq)
## sample       33.4341  2  5.494e-08 ***
## type          6.2475  2    0.04399 *
## sample:type   7.8459  4    0.09739 .
## ---
## Signif. codes:  0 '***' 0.001 '**' 0.01 '*' 0.05 '.' 0.1 ' ' 1
```

```
plot(mod3) #plot standardized residuals against fitted
```

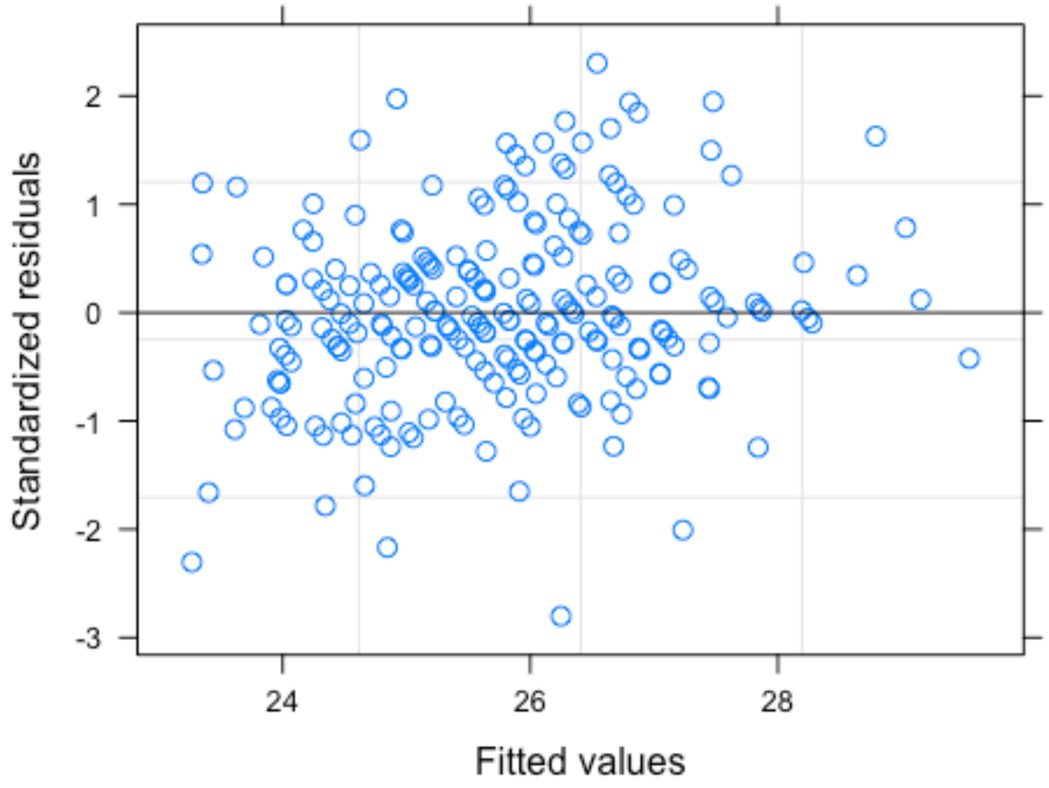


```
#test assumptions
hist(residuals(mod3))
```

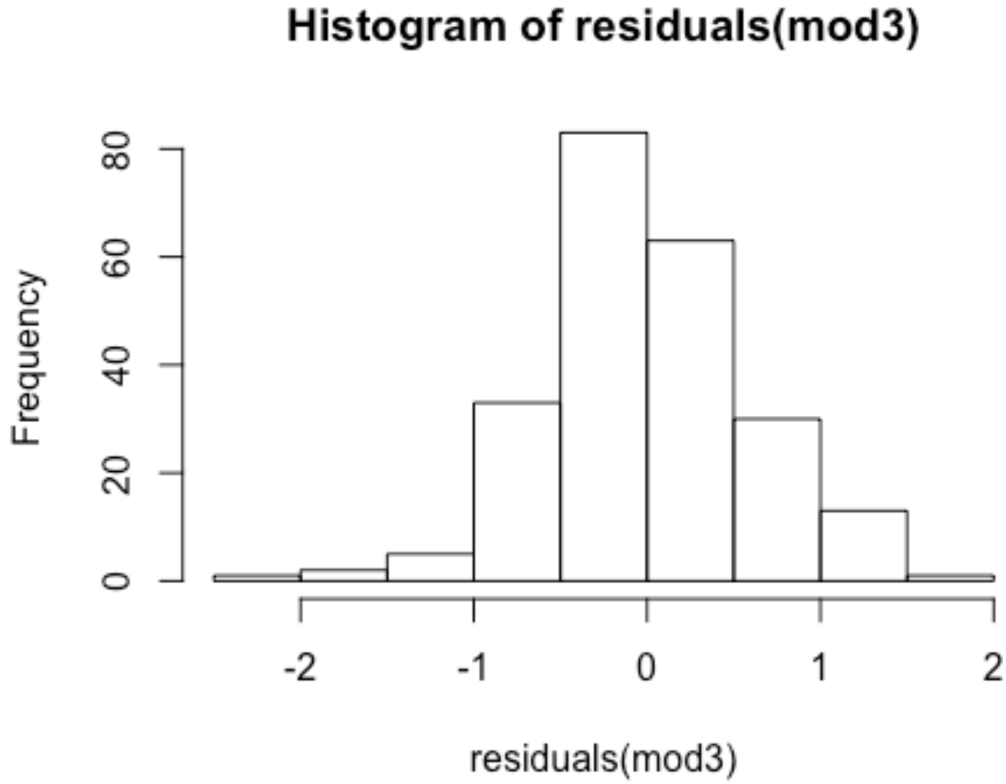


```
qqnorm(residuals(mod3))
```

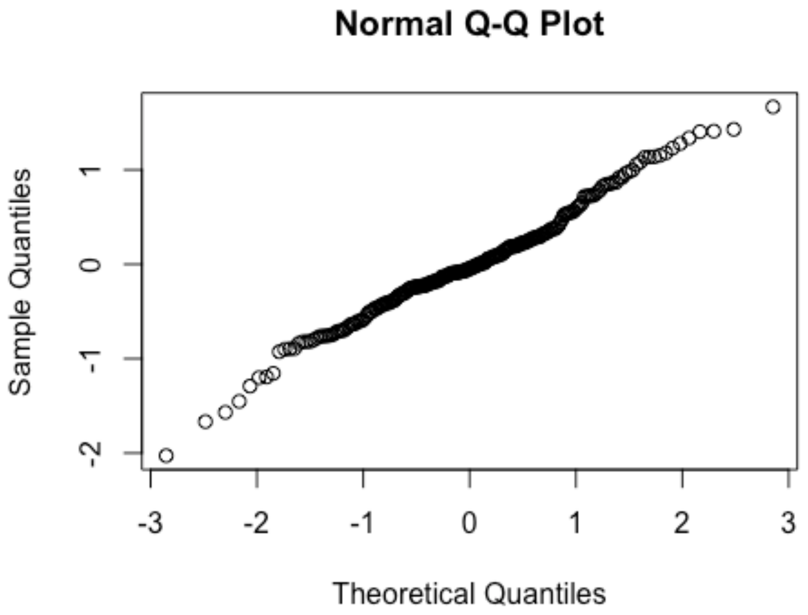

```
shapiro.test(residuals(mod3)) # slight but significant departure from normali
ty

##
##  Shapiro-Wilk normality test
##
## data:  residuals(mod3)
## W = 0.98733, p-value = 0.03859

#due to interaction term woith P< 0.20 and small sample size, analyze pre-pea
k, peak, and post-peak frequencies separately
#compare sex/age groups only for peak frequencies
mod4 <- lme(freq~type, random=~1|id/boutID, data=peak, na.action=na.omit)
summary(mod4)

## Linear mixed-effects model fit by REML
##  Data: peak
##        AIC      BIC    logLik
##   247.3062 261.3691 -117.6531
##
## Random effects:
##  Formula: ~1 | id
##         (Intercept)
## StdDev:    0.922801
##
##  Formula: ~1 | boutID %in% id
##         (Intercept)  Residual
## StdDev:   0.7312362 0.3308566
##
## Fixed effects: freq ~ type
##                  Value Std.Error DF  t-value p-value
## (Intercept)  26.141878 0.5068072 47 51.58150  0.0000
## typejuvenile  0.195135 0.5857460 30  0.33314  0.7413
## typemale     -0.557296 0.5779493 30 -0.96426  0.3426
##  Correlation:
##              (Intr) typjvn
## typejuvenile -0.865
## typemale     -0.877  0.759
##
## Standardized Within-Group Residuals:
##           Min            Q1           Med            Q3           Max
## -0.9640219287 -0.2312873754 -0.0004187539  0.2374871489  0.8826258424
##
## Number of Observations: 80
## Number of Groups:
##             id boutID %in% id
##             33             80

Anova(mod4)
```

```
## Analysis of Deviance Table (Type II tests)
##
## Response: freq
##       Chisq Df Pr(>Chisq)
## type 3.6016  2     0.1652

#test assumptions
hist(residuals(mod4))
```

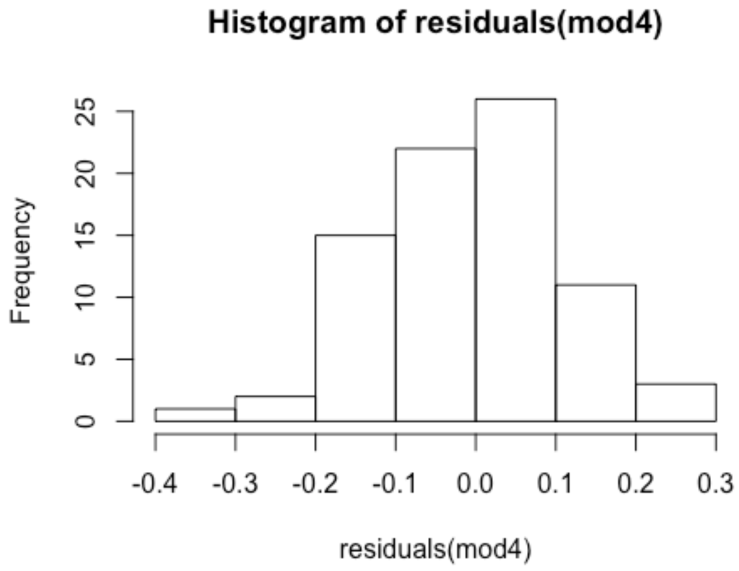


```
qqnorm(residuals(mod4))
```

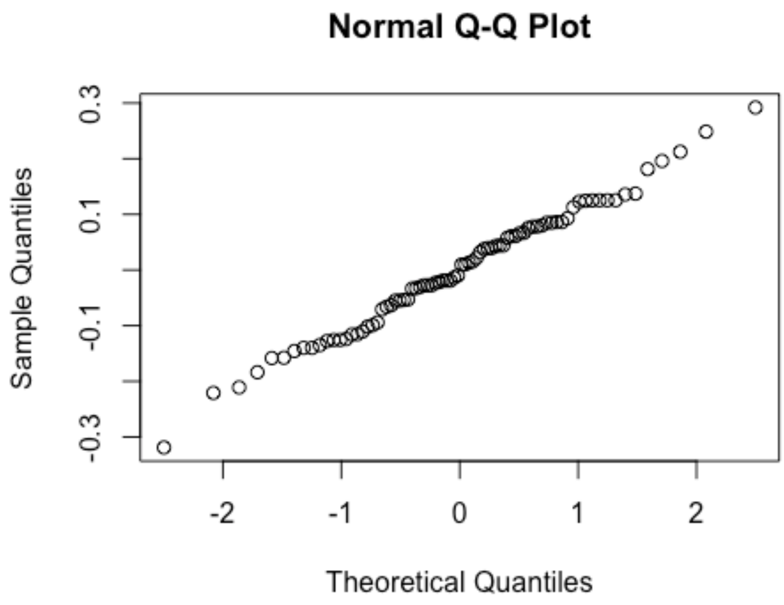

```
shapiro.test(residuals(mod4))

##
##  Shapiro-Wilk normality test
##
## data:  residuals(mod4)
## W = 0.99178, p-value = 0.895

#compare sex/age groups for pre- and post-peak frequencies
pre <- subset(rattle, sample=='pre')
post <- subset(rattle, sample=='post')
mod5 <- lme(freq~type, random=~1|id/boutID, data=pre, na.action=na.omit)
mod6 <- lme(freq~type, random=~1|id/boutID, data=post, na.action=na.omit)
Anova(mod5)

## Analysis of Deviance Table (Type II tests)
##
## Response: freq
##       Chisq Df Pr(>Chisq)
## type 8.9387  2    0.01145 *
## ---
## Signif. codes:  0 '***' 0.001 '**' 0.01 '*' 0.05 '.' 0.1 ' ' 1

Anova(mod6)

## Analysis of Deviance Table (Type II tests)
##
## Response: freq
##       Chisq Df Pr(>Chisq)
## type 6.3647  2    0.04149 *
## ---
## Signif. codes:  0 '***' 0.001 '**' 0.01 '*' 0.05 '.' 0.1 ' ' 1

summary(glht(mod5, linfct=mcp(type='Tukey')))

##
##   Simultaneous Tests for General Linear Hypotheses
##
## Multiple Comparisons of Means: Tukey Contrasts
##
##
## Fit: lme.formula(fixed = freq ~ type, data = pre, random = ~1 | id/boutID,
##     na.action = na.omit)
##
## Linear Hypotheses:
##                        Estimate Std. Error z value Pr(>|z|)
## juvenile - female == 0  -1.0115     0.7980  -1.268   0.4032
## male - female == 0      -1.9872     0.7829  -2.538   0.0282 *
## male - juvenile == 0    -0.9756     0.4494  -2.171   0.0722 .
## ---
```

```
## Signif. codes:  0 '***' 0.001 '**' 0.01 '*' 0.05 '.' 0.1 ' ' 1
## (Adjusted p values reported -- single-step method)
```

```
summary(glht(mod6, linfct=mcp(type='Tukey')))
```

```
##
##   Simultaneous Tests for General Linear Hypotheses
##
## Multiple Comparisons of Means: Tukey Contrasts
##
##
## Fit: lme.formula(fixed = freq ~ type, data = post, random = ~1 | id/boutID
,
##     na.action = na.omit)
##
## Linear Hypotheses:
##                           Estimate Std. Error z value Pr(>|z|)
## juvenile - female == 0  -0.6785     0.8729  -0.777   0.7100
## male - female == 0      -1.7081     0.8617  -1.982   0.1113
## male - juvenile == 0    -1.0296     0.5050  -2.039   0.0982 .
## ---
## Signif. codes:  0 '***' 0.001 '**' 0.01 '*' 0.05 '.' 0.1 ' ' 1
## (Adjusted p values reported -- single-step method)
```

```
shapiro.test(residuals(mod5))
```

```
##
##  Shapiro-Wilk normality test
##
## data:  residuals(mod5)
## W = 0.95607, p-value = 0.01246
```

```
shapiro.test(residuals(mod6))
```

```
##
##  Shapiro-Wilk normality test
##
## data:  residuals(mod6)
## W = 0.99061, p-value = 0.8428
```

Peacock train growth analysis

Details are reported in the supplement to show that the linear approximation that we used was reasonable, and that different feather growth models give very similar growth rates.

```
growth <- growth[with(growth,order(traindate)),] #sort train length data by d
ate

# lognormal model fit
```

```
lognormal <- lm(train~log(traindate), data=growth)
summary(lognormal)

##
## Call:
## lm(formula = train ~ log(traindate), data = growth)
##
## Residuals:
##     Min      1Q  Median      3Q     Max
## -18.725  -5.999  -1.661   6.883  25.197
##
## Coefficients:
##                Estimate Std. Error t value Pr(>|t|)
## (Intercept)      -5.125     12.739  -0.402    0.688
## log(traindate)   31.914      3.060  10.429   <2e-16 ***
## ---
## Signif. codes:  0 '***' 0.001 '**' 0.01 '*' 0.05 '.' 0.1 ' ' 1
##
## Residual standard error: 9.158 on 86 degrees of freedom
## Multiple R-squared:  0.5584, Adjusted R-squared:  0.5533
## F-statistic: 108.8 on 1 and 86 DF,  p-value: < 2.2e-16

# gompertz model fit
getInitial(train~SSgompertz(log(growth$traindate), 150, 30, 0.3), data=growth
)

##         150          30         0.3
## 158.1556593  34.0956349   0.2912793

gompertz <- nls(train~SSgompertz(log(traindate), Asym, b2, b3), data=growth,
start=c(Asym=150, b2=30, b3=0.3))
summary(gompertz)

##
## Formula: train ~ SSgompertz(log(traindate), Asym, b2, b3)
##
## Parameters:
##      Estimate Std. Error t value Pr(>|t|)
## Asym 171.4075    28.8141   5.949 5.84e-08 ***
## b2    10.7980    16.3206   0.662   0.5100
## b3     0.4181     0.2118   1.974   0.0516 .
## ---
## Signif. codes:  0 '***' 0.001 '**' 0.01 '*' 0.05 '.' 0.1 ' ' 1
##
## Residual standard error: 9.14 on 85 degrees of freedom
##
## Number of iterations to convergence: 5
## Achieved convergence tolerance: 5.899e-06

# logistic (3 parameter) model fit
getInitial(train~SSlogis(growth$traindate, 144, 13, 23), data=growth)
```

```
##       144         13         23
## 144.03592  12.95408  23.37796
```

```
logistic3 <- nls(train~SSlogis(traindate, Asym, xmid, scal), data=growth, sta
rt=c(Asym=140, xmid=10, scal=20))
summary(logistic3)
```

```
##
## Formula: train ~ SSlogis(traindate, Asym, xmid, scal)
##
## Parameters:
##      Estimate Std. Error t value Pr(>|t|)
## Asym  146.799      5.016  29.265  < 2e-16 ***
## xmid    7.468      7.776   0.960 0.339582
## scal   28.689      8.108   3.538 0.000655 ***
## ---
## Signif. codes:  0 '***' 0.001 '**' 0.01 '*' 0.05 '.' 0.1 ' ' 1
##
## Residual standard error: 9.16 on 85 degrees of freedom
##
## Number of iterations to convergence: 4
## Achieved convergence tolerance: 2.168e-07
```

```
par(mfrow=c(1,3))
plot(train~traindate, growth, type='p');points(predict(lognormal)~growth$trai
ndate, type='l')
plot(train~traindate, growth, type='p');points(predict(gompertz)~growth$train
date, type='l')
plot(train~traindate, growth, type='p');points(predict(logistic3)~growth$trai
ndate, type='l')
```

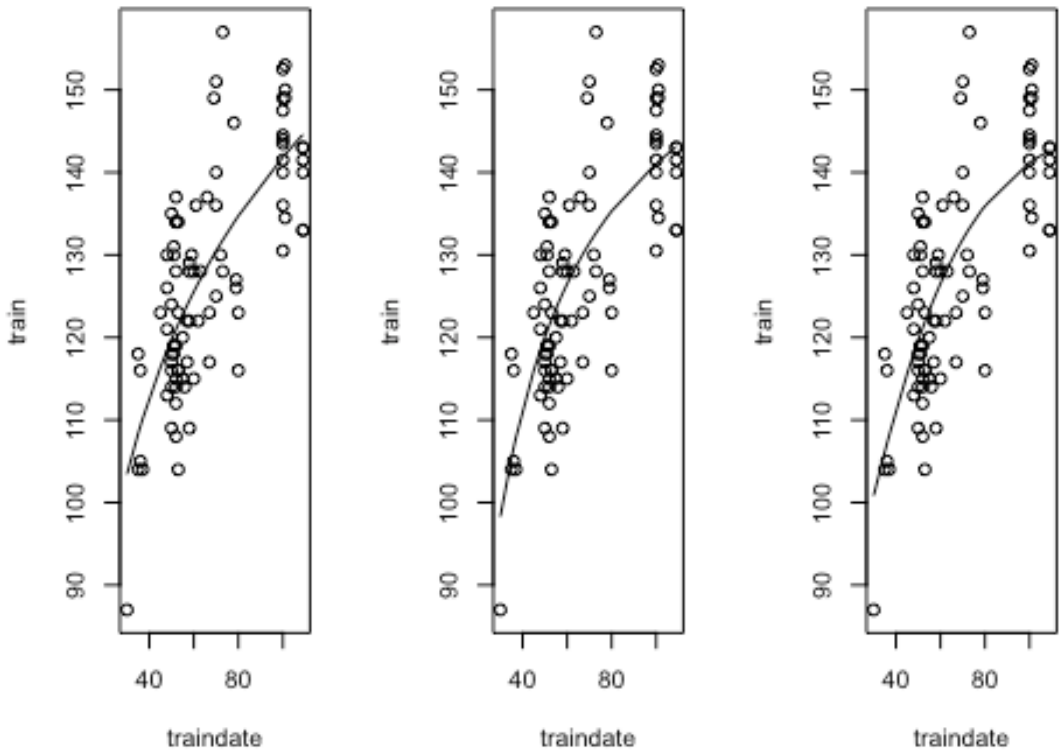


```
# predicted feather growth cm/day over days 69-80 (linear approximation)
unname((predict(lognormal, newdata=data.frame(traindate=c(69,80)))[2]-predict
(lognormal, newdata=data.frame(traindate=c(69,80)))[1])/(80-69))

## [1] 0.4291619

unname((predict(gompertz, newdata=data.frame(traindate=c(69,80)))[2]-predict(
gompertz, newdata=data.frame(traindate=c(69,80)))[1])/(80-69))

## [1] 0.394031

unname((predict(logistic3, newdata=data.frame(traindate=c(69,80)))[2]-predict
(logistic3, newdata=data.frame(traindate=c(69,80)))[1])/(80-69))

## [1] 0.4125708

# bootstrapping the logistic fit to get CI
myfun <- function(x, d){
    fmBoot <- nls(train~SSlogis(traindate, Asym, xmid, scal), data=x[d,], sta
rt=c(Asym=140, xmid=10, scal=20), control=list(maxiter=100,warnOnly=T))
    return((predict(fmBoot, newdata=data.frame(traindate=c(69,80)))[2]-predic
t(fmBoot, newdata=data.frame(traindate=c(69,80)))[1])/(80-69))
}
set.seed(101)
```

```
myboot <- boot(growth, myfun, 10000);(myboot) # 10,000 replicates; result SE
= 0.04652229

##
## ORDINARY NONPARAMETRIC BOOTSTRAP
##
##
## Call:
## boot(data = growth, statistic = myfun, R = 10000)
##
##
## Bootstrap Statistics :
##      original       bias    std. error
## t1* 0.4125708 -0.003430621  0.04652229

boot.ci(myboot, type='bca') #result: 95% CI is 0.3168,  0.49960

## BOOTSTRAP CONFIDENCE INTERVAL CALCULATIONS
## Based on 10000 bootstrap replicates
##
## CALL :
## boot.ci(boot.out = myboot, type = "bca")
##
## Intervals :
## Level       BCa
## 95%   ( 0.3168,  0.4996 )
## Calculations and Intervals on Original Scale
```

Factors associated with peacock vibration frequency

Linear mixed effects models to predict feather vibration freq uency during train-rattling display

```
# calculate train length on date of display, corrected for train growth of 0.
41 cm/day relative to date train was measured
rattle$trainLcor <- rattle$trainL + 0.41 * (rattle$displaydateDOY-rattle$trai
ndateDOY)

# evaluate 13 candidate models: one is intercept-only (null model), other mod
els have either no morphological variables or only 1 of the 3 morphological v
ariables, as well as day+temp, day+time, or day alone. All models except the
intercept-only model include sample period and day of the year

mod.full <- lme(freq~sample+timeH+displaydateDOY+trainLcor+rectrixL+eyespots+
temperatureC, random=~1|id/boutID, data=rattle[!is.na(rattle$freq)&!is.na(rat
tle$trainL),], method='ML')
summary(mod.full)

## Linear mixed-effects model fit by maximum likelihood
##  Data: rattle[!is.na(rattle$freq) & !is.na(rattle$trainL), ]
```

```
##        AIC      BIC    logLik
##   287.6003 319.333 -131.8002
##
## Random effects:
##  Formula: ~1 | id
##         (Intercept)
## StdDev:    0.220031
##
##  Formula: ~1 | boutID %in% id
##         (Intercept)  Residual
## StdDev:   0.6656693 0.6757033
##
## Fixed effects: freq ~ sample + timeH + displaydateDOY + trainLcor + rectri
xL +      eyespots + temperatureC
##                     Value Std.Error DF   t-value p-value
## (Intercept)    23.228195  9.479586 67  2.450339  0.0169
## samplepost     -0.694167  0.169002 67 -4.107447  0.0001
## samplepre      -0.073396  0.170668 67 -0.430053  0.6685
## timeH           0.126117  0.053640 19  2.351175  0.0297
## displaydateDOY -0.131239  0.063985 19 -2.051071  0.0543
## trainLcor       0.021627  0.021457 19  1.007923  0.3262
## rectrixL        0.047214  0.094644  9  0.498861  0.6298
## eyespots        0.047549  0.032653  9  1.456189  0.1793
## temperatureC   -0.066031  0.067883 19 -0.972725  0.3429
##  Correlation:
##                (Intr) smplps smplpr timeH  dspDOY trnLcr rctrxL eyspts
## samplepost     -0.009
## samplepre      -0.010  0.495
## timeH           0.090  0.000 -0.003
## displaydateDOY -0.799  0.000  0.004 -0.282
## trainLcor       0.626  0.000 -0.005 -0.106 -0.359
## rectrixL       -0.735  0.000  0.005  0.095  0.417 -0.776
## eyespots       -0.654  0.000 -0.003  0.123  0.190 -0.622  0.427
## temperatureC   -0.562  0.000  0.000 -0.592  0.701 -0.248  0.251  0.082
##
## Standardized Within-Group Residuals:
##         Min          Q1         Med          Q3         Max
## -1.84723807 -0.67381081 -0.05992619  0.56983047  2.64739447
##
## Number of Observations: 104
## Number of Groups:
##             id boutID %in% id
##             12             35
```

```
#compare and evaluate 13 candidate models
(mods <- dredge(mod.full, subset=
(displaydateDOY&sample&!timeH&temperatureC& trainLcor&!eyespots&!rectrixL)|
(displaydateDOY&sample&!timeH&temperatureC& !trainLcor&eyespots&!rectrixL)|
(displaydateDOY&sample&!timeH&temperatureC& !trainLcor&!eyespots&rectrixL)|
(displaydateDOY&sample&!timeH&temperatureC& !trainLcor&!eyespots&!rectrixL)|
```

```
(displaydateDOY&sample&timeH&!temperatureC& trainLcor&!eyespots&!rectrixL)|
(displaydateDOY&sample&timeH&!temperatureC& !trainLcor&eyespots&!rectrixL)|
(displaydateDOY&sample&timeH&!temperatureC& !trainLcor&!eyespots&rectrixL)|
(displaydateDOY&sample&timeH&!temperatureC& !trainLcor&!eyespots&!rectrixL)|
(displaydateDOY&sample&!timeH&!temperatureC& trainLcor&!eyespots&!rectrixL)|
(displaydateDOY&sample&!timeH&!temperatureC& !trainLcor&eyespots&!rectrixL)|
(displaydateDOY&sample&!timeH&!temperatureC& !trainLcor&!eyespots&rectrixL)|
(displaydateDOY&sample&!timeH&!temperatureC& !trainLcor&!eyespots&!rectrixL)|
(!displaydateDOY&!sample&!timeH&!temperatureC& !trainLcor&!eyespots&!rectrixL
)))

## Fixed term is "(Intercept)"

## Global model call: lme.formula(fixed = freq ~ sample + timeH + displaydate
DOY +
##     trainLcor + rectrixL + eyespots + temperatureC, data = rattle[!is.na(r
attle$freq) &
##     !is.na(rattle$trainL), ], random = ~1 | id/boutID, method = "ML")
## ---
## Model selection table
##     (Intrc)    dsDOY   eyspt  rctrL sampl   tmprC   timeH   trnLc df
## 106   26.98 -0.09684                   +         0.08391 0.03778  9
## 74    28.82 -0.11230                   +                 0.04017  8
## 44    20.26 -0.08557 0.07219           +         0.10050          9
## 46    22.47 -0.06903         0.1460    +         0.10870          9
## 90    27.36 -0.09760                   + 0.02116         0.03938  9
## 42    30.59 -0.07949                   +         0.10070          8
## 12    23.16 -0.10300 0.06963           +                          8
## 10    33.31 -0.09944                   +                          7
## 14    26.41 -0.09013         0.1249    +                          8
## 28    20.39 -0.07581 0.06873           + 0.04007                  9
## 30    21.79 -0.05105         0.1342    + 0.05636                  9
## 26    30.15 -0.06984                   + 0.04324                  8
## 1     25.36                                                       4
##       logLik  AICc delta weight
## 106 -133.445 286.8  0.00  0.387
## 74  -135.298 288.1  1.31  0.201
## 44  -134.504 288.9  2.12  0.134
## 46  -135.150 290.2  3.41  0.070
## 90  -135.221 290.4  3.55  0.065
## 42  -136.870 291.3  4.45  0.042
## 12  -136.966 291.4  4.64  0.038
## 10  -138.959 293.1  6.28  0.017
## 14  -137.819 293.2  6.35  0.016
## 28  -136.710 293.3  6.53  0.015
## 30  -137.347 294.6  7.80  0.008
## 26  -138.692 294.9  8.09  0.007
## 1   -150.907 310.2 23.41  0.000
## Models ranked by AICc(x)
```

```
## Random terms (all models):
## '1 | id', '1 | boutID %in% id'

avmods <- model.avg(mods,subset = delta < 2)
summary(avmods)

##
## Call:
## model.avg.model.selection(object = mods, subset = delta < 2)
##
## Component model call:
## lme.formula(fixed = freq ~ <2 unique rhs>, data =
##      rattle[!is.na(rattle$freq) & !is.na(rattle$trainL), ], random = ~1
##      | id/boutID, method = ML)
##
## Component models:
##      df  logLik   AICc delta weight
## 1234  9 -133.45 286.81  0.00   0.66
## 124   8 -135.30 288.11  1.31   0.34
##
## Term codes:
## displaydateDOY         sample           timeH      trainLcor
##              1              2               3              4
##
## Model-averaged coefficients:
## (full average)
##                Estimate Std. Error Adjusted SE z value Pr(>|z|)
## (Intercept)    27.60775    4.01026     4.08053   6.766  < 2e-16 ***
## displaydateDOY -0.10213    0.04476     0.04751   2.150  0.03159 *
## samplepost     -0.69417    0.16589     0.16894   4.109 3.97e-05 ***
## samplepre      -0.07005    0.16754     0.17062   0.411  0.68139
## timeH           0.05519    0.05298     0.05449   1.013  0.31115
## trainLcor       0.03860    0.01281     0.01362   2.835  0.00458 **
##
## (conditional average)
##                Estimate Std. Error Adjusted SE z value Pr(>|z|)
## (Intercept)    27.60775    4.01026     4.08053   6.766  < 2e-16 ***
## displaydateDOY -0.10213    0.04476     0.04751   2.150  0.03159 *
## samplepost     -0.69417    0.16589     0.16894   4.109 3.97e-05 ***
## samplepre      -0.07005    0.16754     0.17062   0.411  0.68139
## timeH           0.08391    0.04311     0.04588   1.829  0.06742 .
## trainLcor       0.03860    0.01281     0.01362   2.835  0.00458 **
## ---
## Signif. codes:  0 '***' 0.001 '**' 0.01 '*' 0.05 '.' 0.1 ' ' 1
##
## Relative variable importance:
##                      displaydateDOY sample trainLcor timeH
## Importance:          1.00           1.00   1.00      0.66
## N containing models:    2              2      2         1
```

```
confint(avmods)

##                       2.5 %       97.5 %
## (Intercept)    19.610061686 35.605440629
## displaydateDOY -0.195246821 -0.009009527
## samplepost     -1.025279934 -0.363053701
## samplepre      -0.404466667  0.264361725
## timeH          -0.006015104  0.173826431
## trainLcor       0.011913274  0.065284719

#same but this time with standardized predictors
(modsbeta <- dredge(mod.full, beta = "sd", subset=
(displaydateDOY&sample&!timeH&temperatureC& trainLcor&!eyespots&!rectrixL)|
(displaydateDOY&sample&!timeH&temperatureC& !trainLcor&eyespots&!rectrixL)|
(displaydateDOY&sample&!timeH&temperatureC& !trainLcor&!eyespots&rectrixL)|
(displaydateDOY&sample&!timeH&temperatureC& !trainLcor&!eyespots&!rectrixL)|
(displaydateDOY&sample&timeH&!temperatureC& trainLcor&!eyespots&!rectrixL)|
(displaydateDOY&sample&timeH&!temperatureC& !trainLcor&eyespots&!rectrixL)|
(displaydateDOY&sample&timeH&!temperatureC& !trainLcor&!eyespots&rectrixL)|
(displaydateDOY&sample&timeH&!temperatureC& !trainLcor&!eyespots&!rectrixL)|
(displaydateDOY&sample&!timeH&!temperatureC& trainLcor&!eyespots&!rectrixL)|
(displaydateDOY&sample&!timeH&!temperatureC& !trainLcor&eyespots&!rectrixL)|
(displaydateDOY&sample&!timeH&!temperatureC& !trainLcor&!eyespots&rectrixL)|
(displaydateDOY&sample&!timeH&!temperatureC& !trainLcor&!eyespots&!rectrixL)|
(!displaydateDOY&!sample&!timeH&!temperatureC& !trainLcor&!eyespots&!rectrixL
)))

## Fixed term is "(Intercept)"

## Global model call: lme.formula(fixed = freq ~ sample + timeH + displaydate
DOY +
##     trainLcor + rectrixL + eyespots + temperatureC, data = rattle[!is.na(r
attle$freq) &
##     !is.na(rattle$trainL), ], random = ~1 | id/boutID, method = "ML")
## ---
## Model selection table
##     (Intrc)   dsDOY  eyspt  rctrL sampl   tmprC  timeH  trnLc df   logLik
## 106       0 -0.2482                   +         0.2349 0.3877  9 -133.445
## 74        0 -0.2878                   +                0.4122  8 -135.298
## 44        0 -0.2193 0.3374            +         0.2814         9 -134.504
## 46        0 -0.1769        0.3016     +         0.3044         9 -135.150
## 90        0 -0.2501                   + 0.05987        0.4041  9 -135.221
## 42        0 -0.2037                   +         0.2820         8 -136.870
## 12        0 -0.2640 0.3255            +                        8 -136.966
## 10        0 -0.2549                   +                        7 -138.959
## 14        0 -0.2310        0.2581     +                        8 -137.819
## 28        0 -0.1943 0.3213            + 0.11340                9 -136.710
## 30        0 -0.1309        0.2771     + 0.15950                9 -137.347
## 26        0 -0.1790                   + 0.12240                8 -138.692
## 1         0                                                    4 -150.907
##      AICc delta weight
```

```
## 106 286.8  0.00  0.387
## 74  288.1  1.31  0.201
## 44  288.9  2.12  0.134
## 46  290.2  3.41  0.070
## 90  290.4  3.55  0.065
## 42  291.3  4.45  0.042
## 12  291.4  4.64  0.038
## 10  293.1  6.28  0.017
## 14  293.2  6.35  0.016
## 28  293.3  6.53  0.015
## 30  294.6  7.80  0.008
## 26  294.9  8.09  0.007
## 1   310.2 23.41  0.000
## Models ranked by AICc(x)
## Random terms (all models):
## '1 | id', '1 | boutID %in% id'
```

```
avmodsbeta <- model.avg(modsbeta,subset = delta < 2)
summary(avmodsbeta)
```

```
##
## Call:
## model.avg.model.selection(object = modsbeta, subset = delta <
##     2)
##
## Component model call:
## lme.formula(fixed = freq ~ <2 unique rhs>, data =
##      rattle[!is.na(rattle$freq) & !is.na(rattle$trainL), ], random = ~1
##      | id/boutID, method = ML)
##
## Component models:
##      df  logLik   AICc delta weight
## 1234  9 -133.45 286.81  0.00   0.66
## 124   8 -135.30 288.11  1.31   0.34
##
## Term codes:
## displaydateDOY         sample          timeH      trainLcor
##              1              2              3              4
##
## Model-averaged coefficients:
## (full average)
##                Estimate Std. Error Adjusted SE z value Pr(>|z|)
## (Intercept)     0.00000    0.00000     0.00000      NA       NA
## displaydateDOY -0.26176    0.11472     0.12177   2.150  0.03159 *
## samplepost     -0.25484    0.06090     0.06202   4.109 3.97e-05 ***
## samplepre      -0.02553    0.06106     0.06218   0.411  0.68139
## timeH           0.15448    0.14830     0.15253   1.013  0.31115
## trainLcor       0.39609    0.13147     0.13972   2.835  0.00458 **
##
## (conditional average)
```

```
##                   Estimate Std. Error Adjusted SE z value Pr(>|z|)
## (Intercept)        0.00000    0.00000     0.00000      NA       NA
## displaydateDOY   -0.26176    0.11472     0.12177   2.150  0.03159 *
## samplepost       -0.25484    0.06090     0.06202   4.109 3.97e-05 ***
## samplepre        -0.02553    0.06106     0.06218   0.411  0.68139
## timeH             0.23486    0.12066     0.12842   1.829  0.06742 .
## trainLcor         0.39609    0.13147     0.13972   2.835  0.00458 **
## ---
## Signif. codes:  0 '***' 0.001 '**' 0.01 '*' 0.05 '.' 0.1 ' ' 1
##
## Relative variable importance:
##                      displaydateDOY sample trainLcor timeH
## Importance:          1.00           1.00   1.00      0.66
## N containing models:    2              2      2         1
```

```
confint(avmodsbeta)
```

```
##                        2.5 %      97.5 %
## (Intercept)        0.00000000  0.00000000
## displaydateDOY   -0.50043170 -0.02309207
## samplepost       -0.37640323 -0.13328515
## samplepre        -0.14740884  0.09634726
## timeH            -0.01683685  0.48655675
## trainLcor         0.12225106  0.66993556
```

```
#best-fitting model
mod.best <- lmer(freq~displaydateDOY+sample+timeH+trainLcor+(1|id/boutID), da
ta=rattle[!is.na(rattle$freq)&!is.na(rattle$trainL),]) # best model
plot(mod.best) #plot stadardized residuals against fitted
```

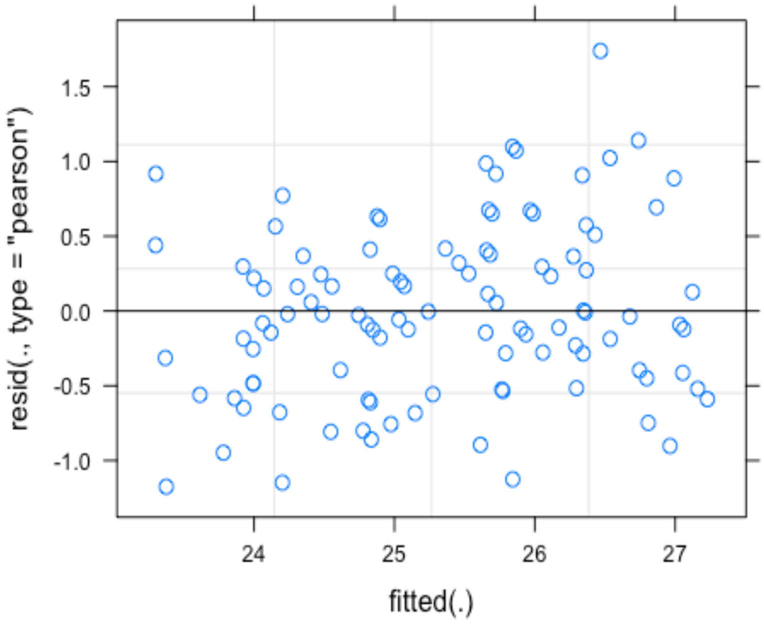

```
summary(mod.best)

## Linear mixed model fit by REML ['lmerMod']
## Formula:
## freq ~ displaydateDOY + sample + timeH + trainLcor + (1 | id/boutID)
##    Data: rattle[!is.na(rattle$freq) & !is.na(rattle$trainL), ]
##
## REML criterion at convergence: 288.1
##
## Scaled residuals:
##      Min       1Q   Median       3Q      Max
## -1.71583 -0.66921 -0.04727  0.54083  2.54046
##
## Random effects:
##  Groups    Name        Variance Std.Dev.
##  boutID:id (Intercept) 0.4923   0.7017
##  id        (Intercept) 0.1920   0.4382
##  Residual              0.4687   0.6846
## Number of obs: 104, groups:  boutID:id, 35; id, 12
##
## Fixed effects:
##                Estimate Std. Error t value
## (Intercept)    26.61626    3.97906   6.689
## displaydateDOY -0.09255    0.04400  -2.103
## samplepost     -0.69417    0.16366  -4.242
## samplepre      -0.06935    0.16529  -0.420
## timeH           0.08750    0.04519   1.936
## trainLcor       0.03769    0.01401   2.691
##
## Correlation of Fixed Effects:
##             (Intr) dspDOY smplps smplpr timeH
## displydtDOY -0.871
## samplepost  -0.021  0.000
## samplepre   -0.022  0.004  0.495
## timeH       -0.240  0.169  0.000 -0.003
## trainLcor   -0.458 -0.015  0.000 -0.003 -0.076

shapiro.test(residuals(mod.best))

##
##  Shapiro-Wilk normality test
##
## data:  residuals(mod.best)
## W = 0.98886, p-value = 0.5453

#calulate R^2 for best fitting model
r.squaredGLMM(mod.best)

##       R2m       R2c
## 0.3521577 0.7366622
```

```
mod.TLonly <- lmer(freq~trainLcor+(1|id/boutID), data=rattle[!is.na(rattle$fr
eq)&!is.na(rattle$trainL),])  # model with train length only
r.squaredGLMM(mod.TLonly)
```

```
##       R2m       R2c
## 0.1668576 0.6459990
```

```
#same model as mod.full, above, without correcting for train growth
mod.full2 <- lme(freq~sample+timeH+displaydateDOY+trainL+rectrixL+eyespots+te
mperatureC, random=~1|id/boutID, data=rattle[!is.na(rattle$freq)&!is.na(rattl
e$trainL),], method='ML')

(mods2 <- dredge(mod.full2, subset=
(displaydateDOY&sample&!timeH&temperatureC& trainL&!eyespots&!rectrixL)|
(displaydateDOY&sample&!timeH&temperatureC& !trainL&eyespots&!rectrixL)|
(displaydateDOY&sample&!timeH&temperatureC& !trainL&!eyespots&rectrixL)|
(displaydateDOY&sample&!timeH&temperatureC& !trainL&!eyespots&!rectrixL)|
(displaydateDOY&sample&timeH&!temperatureC& trainL&!eyespots&!rectrixL)|
(displaydateDOY&sample&timeH&!temperatureC& !trainL&eyespots&!rectrixL)|
(displaydateDOY&sample&timeH&!temperatureC& !trainL&!eyespots&rectrixL)|
(displaydateDOY&sample&timeH&!temperatureC& !trainL&!eyespots&!rectrixL)|
(displaydateDOY&sample&!timeH&!temperatureC& trainL&!eyespots&!rectrixL)|
(displaydateDOY&sample&!timeH&!temperatureC& !trainL&eyespots&!rectrixL)|
(displaydateDOY&sample&!timeH&!temperatureC& !trainL&!eyespots&rectrixL)|
(displaydateDOY&sample&!timeH&!temperatureC& !trainL&!eyespots&!rectrixL)|
(!displaydateDOY&!sample&!timeH&!temperatureC& !trainL&!eyespots&!rectrixL)))
```

```
## Fixed term is "(Intercept)"
```

```
## Global model call: lme.formula(fixed = freq ~ sample + timeH + displaydate
DOY +
##     trainL + rectrixL + eyespots + temperatureC, data = rattle[!is.na(ratt
le$freq) &
##     !is.na(rattle$trainL), ], random = ~1 | id/boutID, method = "ML")
## ---
## Model selection table
##     (Intrc)    dsDOY   eyspt  rctrL sampl   tmprC   timeH   tranL df
## 106   25.75 -0.08456                    +         0.08097 0.04041  9
## 74    27.41 -0.09927                    +                 0.04350  8
## 44    20.26 -0.08557 0.07219            +         0.10050          9
## 46    22.47 -0.06903         0.1460     +         0.10870          9
## 90    26.33 -0.08818                    + 0.01610         0.04273  9
## 42    30.59 -0.07949                    +         0.10070          8
## 12    23.16 -0.10300 0.06963            +                          8
## 10    33.31 -0.09944                    +                          7
## 14    26.41 -0.09013         0.1249     +                          8
## 28    20.39 -0.07581 0.06873            + 0.04007                  9
## 30    21.79 -0.05105         0.1342     + 0.05636                  9
## 26    30.15 -0.06984                    + 0.04324                  8
## 1     25.36                                                        4
##       logLik  AICc delta weight
```

```
## 106 -133.519 287.0  0.00  0.363
## 74  -135.224 288.0  1.01  0.219
## 44  -134.504 288.9  1.97  0.135
## 46  -135.150 290.2  3.26  0.071
## 90  -135.181 290.3  3.32  0.069
## 42  -136.870 291.3  4.30  0.042
## 12  -136.966 291.4  4.49  0.038
## 10  -138.959 293.1  6.13  0.017
## 14  -137.819 293.2  6.20  0.016
## 28  -136.710 293.3  6.38  0.015
## 30  -137.347 294.6  7.66  0.008
## 26  -138.692 294.9  7.95  0.007
## 1   -150.907 310.2 23.27  0.000
## Models ranked by AICc(x)
## Random terms (all models):
## '1 | id', '1 | boutID %in% id'
```

SUMMARY: model that does not correct for train growth (mods2) gives same results as model correcting for train growth except that in mods2 analysis, there are 3 instead of 2 top models and the third ranked model includes the number of eyespots as a predictor
